# Nanoscale capacitance spectroscopy based on multifrequency electrostatic force microscopy

**DOI:** 10.3762/bjnano.16.49

**Published:** 2025-05-08

**Authors:** Pascal N Rohrbeck, Lukas D Cavar, Franjo Weber, Peter G Reichel, Mara Niebling, Stefan A L Weber

**Affiliations:** 1 Max Planck Institute for Polymer Research, Ackermannweg 10, 55128 Mainz, Germany; 2 Department of Chemistry, University of Mainz, Duesbergweg 10-14, 55128 Mainz, Germany; 3 Department of Physics, University of Mainz, Staudingerweg 7, 55128 Mainz, Germany; 4 Institute for Photovoltaics, University of Stuttgart, Pfaffenwaldring 47, 70569 Stuttgart, Germany

**Keywords:** atomic force microscopy, capacitance gradients, dielectric constant, dielectric spectroscopy, heterodyne frequency mixing, Kelvin probe force microscopy, multifrequency AFM, quantitative force spectroscopy, scanning capacitance force microscopy

## Abstract

We present multifrequency heterodyne electrostatic force microscopy (MFH-EFM) as a novel electrostatic force microscopy method for nanoscale capacitance characterization at arbitrary frequencies above the second cantilever resonance. Besides a high spatial resolution, the key advantage of the multifrequency approach of MFH-EFM is that it measures the second-order capacitance gradient at almost arbitrary frequencies, enabling the measurement of the local dielectric function over a wide range of frequencies. We demonstrate the reliable operation of MFH-EFM using standard atomic force microscopy equipment plus an external lock-in amplifier up to a frequency of 5 MHz, which can in principle be extended to gigahertz frequencies and beyond. Our results show a significant reduction of signal background from long-range electrostatic interactions, resulting in highly localized measurements. Combined with refined tip–sample capacitance models, MFH-EFM will enhance the precision of quantitative studies on dielectric effects in nanoscale systems across materials science, biology, and nanotechnology, complementing established methods in the field.

## Introduction

Technological progress in fields including electronics, energy storage, photonics, and biomedical devices would not have been possible without the development of new materials. Progress in these areas requires a detailed understanding of material properties, particularly at the nanoscale, where phenomena such as quantum confinement, interface effects, and defect dynamics play a critical role. Innovations in characterization techniques have enabled researchers to explore these properties with unprecedented precision, paving the way for the design of materials with tailored functionalities [1–6].

Dielectric properties are fundamental for understanding the behavior and performance of various material systems, as they directly influence charge storage, polarization, and energy dissipation mechanisms. For instance, in microelectronic devices, high-κ dielectric materials such as HfO_2_ and ZrO_2_ are critical for minimizing leakage currents and enhancing gate capacitance in transistors [7–9]. In energy storage systems, the dielectric constants of polymer–ceramic composites determine the efficiency and reliability of capacitors [10]. Similarly, in next-generation photovoltaic devices, the dielectric properties of absorber layers, such as lead-halide perovskites, affect carrier recombination and electric field distribution, thereby influencing power conversion efficiency [11].

At the nanoscale, the importance of dielectric properties becomes even more pronounced. Many advanced materials exhibit nanoscale structural heterogeneity, where quantum confinement, phase composition, and interfacial effects cause significant deviations in dielectric behavior compared to bulk materials [12–13]. These nanoscale variations influence key properties such as charge transport, polarization dynamics, and defect distributions, directly impacting the performance of microelectronic and energy systems [14–15]. Understanding these effects requires correlating nanoscale dielectric properties with structural and morphological features.

Scanning probe techniques have revolutionized nanoscale material characterization. Since the invention of scanning tunneling microscopy (STM) [16] and atomic force microscopy (AFM) [17], various electric force-based methods, called electrostatic force microscopy (EFM) methods, have emerged to study materials such as perovskite solar cells [18–20] and Li-ion batteries [21–23]. AFM enables simultaneous acquisition of topographic and electronic data by applying AC or DC voltages across the tip–sample gap, allowing for the detection of capacitive forces [24–25] or contact potential difference (CPD) [18]. Its exceptional spatial resolution, ranging from sub-micrometer [24,26] to atomic scales [27–28], makes AFM a powerful tool for nanoscale analysis.

Scanning probe-based capacitance mapping methods can be divided into two categories: Methods measuring the tip–sample capacitance directly are referred to as scanning capacitance microscopy (SCM) [29–54], whereas methods measuring the capacitive tip–sample force are referred to as scanning capacitance force microscopy (SCFM) [24–25,55–73]. Compared to optical ellipsometry or reflectance spectroscopy, SCM and SCFM can map surface properties such as film thickness [35,39] and dielectric constants [35,74], with superior spatial resolution. However, in particular, SCM techniques face limitations due to nonlocal stray capacitances [40] from cantilever, tip cone, and the electrical connection, which hamper precise measurements and decrease resolution [55,61].

The advantage of SCFM methods is that capacitive forces depend on the first- or higher-order capacitance gradients with respect to the tip–sample distance, automatically canceling out the background capacitance caused by electrical connections and – to some degree – by the cantilever and the tip cone [24–25,55–73]. For example, Cherniavskaya et al. and Crider et al. laid the groundwork for EFM-based nanoscale dielectric measurements such as SCFM [68–69]. Generally, EFM methods using higher-order capacitance gradients exhibit superior lateral resolution [75].

An interesting extension of SCM and SCFM is the possibility to vary the electrostatic excitation frequency, enabling broadband dielectric nanospectroscopy experiments. While it is relatively straightforward to measure the frequency-dependent capacitance in SCM [29,54,76–77], force-based SCFM measurements are usually coupled to the cantilever resonances, limiting the available frequency space. Single-pass second-harmonic EFM in the attractive regime has been used to detect the cantilever response at the second harmonic of the electrostatic force (2ω) [68–70,72] generated when Δω_e_ spans the range from 8 kHz to 2 MHz [70]. SCFM in the megahertz regime has been implemented [70–71] as well as a heterodyne-based EFM mode [59,72–73,78]. By using a low-frequency modulation of a high-frequency electrostatic drive, the response can be picked up either via a frequency shift or by an electrostatic response at one of the cantilever’s resonance frequencies. Thus, the dielectric response can be studied at almost arbitrary frequencies. Using this method, Gramse et al. have demonstrated broadband spectroscopy of dielectric layers in air [72] and water [59].

Building on this idea, we propose a novel, multifrequency AFM-based method for nanoscale capacitance characterization at arbitrary frequencies above the second cantilever resonance. Our approach measures the second capacitance gradient, enhancing localization by minimizing stray capacitance contributions [65]. This method enables high-frequency capacitance gradient spectroscopy without requiring specialized equipment beyond a lock-in amplifier (LIA).

The following sections introduce the theoretical framework of multifrequency EFM, demonstrate its resolution enhancement experimentally, and validate its spectroscopic capabilities by measuring nanoscale dielectric properties of microfabricated SiO_2_ samples. Finally, we compare its performance with established techniques through capacitance imaging of a model microcapacitor system and a perfluoroalkyl-alkane F(CF_2_)_14_(CH_2_)_20_H (F14H20) sample.

## Theory

### Multifrequency electrostatic force microscopy

The electrostatic force *F*_ES_ between tip and sample can be understood in terms of the gradient of the energy, *W_C_*, stored in the tip–sample capacitor *C* with respect to the tip–sample separation *z*, as given by


[1] $$F_{\text{ES}}=\frac{\partial W_{C}}{\partial z}=\frac{1}{2}\cdot \frac{\partial C}{\partial z}\cdot V_{\text{tip}-\text{sample}}^{2}\text{,}$$

where *V*_tip−sample_ specifies the electrical voltage across the tip–sample gap. In conventional EFM with single-frequency excitation, *V*_tip−sample_ is given by Equation 2 [18]:


[2] $$V_{\text{ES}}=V_{\text{DC}}-V_{\text{CPD}}+V_{\text{AC}}\cdot \sin\left(\omega_{\text{e}}\cdot t\right),$$

with *V*_DC_ the DC voltage offset applied to the tip, *V*_AC_ the AC voltage amplitude with the frequency ω_AC_ at a certain time *t* and *V*_CPD_ the CPD, which corresponds to the difference in tip and sample work function [18]. Inserting Equation 2 into Equation 1, we obtain the following expression:


[3] $$F_{\text{ES}}=\frac{1}{2}\frac{\partial C}{\partial z}\left({\left(V_{\text{DC}}-V_{\text{CPD}}\right)}^{2}+\frac{V_{\text{AC}}^{2}}{2}\right)$$

[4] $$+\frac{\partial C}{\partial z}\left(V_{\text{DC}}-V_{\text{CPD}}\right)V_{\text{AC}}\sin\left(\omega_{\text{e}}t\right)$$

[5] $$+\frac{\partial C}{\partial z}\frac{V_{\text{AC}}^{2}}{4}\cos\left(2\omega_{\text{e}}t\right).$$

Alongside a static component in Equation 3, the electrostatic force has periodic time-dependent components at frequencies ω_e_ and 2ω_e_, which correspond to Equation 4 and Equation 5, respectively. In the case of an oscillating AFM tip, the tip–sample distance *z* and, thereby, the tip–sample capacitance and its gradients are changing periodically. This periodic fluctuation of the capacitance gradient 
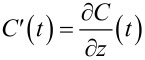
 adds an additional dynamic component to Equations Equation 3–Equation 5. Using a Fourier expansion for the capacitance gradient 
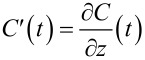
 yields [18]:


[6] $$C\prime \left(t\right)=C\prime \left(z_{0}\right)+{C^{\prime }}^{\prime }\left(z_{0}\right)\cdot A_{\text{m}}\cdot \cos\left(\omega_{\text{m}}t\right)+\ldots$$

By inserting Equation 6 into Equations Equation 3–Equation 5, we find that frequency mixing between *C*′(*t*) and the electrostatic excitation leads to sidebands at frequencies ω_SB,1_ = (ω_m_ ± ω_AC_) and ω_SB,2_ = (ω_m_ ± 2ω_AC_) besides the mechanical oscillation at ω_m_[18]. The amplitude of the first harmonic frequency components is used in conventional amplitude modulation (AM) and sideband or heterodyne Kelvin probe force microscopy (KPFM) [18,26,79]. The second harmonic signals are proportional to the local capacitance gradients, providing information about the local tip–sample capacitance. To ensure a sufficient signal-to-noise ratio, the resulting frequencies should coincide with one of the cantilever’s resonance frequencies, limiting the choice of excitation frequencies.

We can avoid this limitation by using a multifrequency excitation approach. With a double-frequency excitation, we can write the tip–sample voltage as


[7] $$V_{\text{tip}-\text{sample}}=V_{\text{AC,1}}\cdot \sin\left(\omega_{\text{e,1}}t\right)+V_{\text{AC,2}}\cdot \sin\left(\omega_{\text{e,2}}t\right).$$

In the case of two drives with identical amplitude *V*_AC,1_ = *V*_AC,2_ = 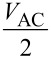, Equation 7 can be rearranged as


[8] $$V_{\text{tip-sample}}=V_{\text{AC}}\cdot \sin\left(\frac{\omega_{\text{e,1}}-\omega_{\text{e,2}}}{2}t\right)\cdot \sin\left(\frac{\omega_{\text{e,1}}+\omega_{\text{e,2}}}{2}t\right).$$

Thus, the waveform can be viewed as a high-frequency oscillation at (ω_e,1_ + ω_e,2_)/2 = ω_mod_/2 with a low-frequency amplitude modulation at frequency (ω_e,1_ − ω_e,2_)/2 = Δω_e_/2. This effect is also known as “beating” and is utilized in the AFM context for example in intermodulation AFM [80–83].

By inserting Equation 6 and Equation 8 in Equations Equation 3–Equation 5, we obtain the full expression for the electrostatic force. Here, we will focus on the DC force component in Equation 3 and set *V*_DC_ − *V*_CPD_ = Δ:


[9] $$F_{\text{DC}}=\frac{1}{2}\left(C\prime +{C^{\prime }}^{\prime }A_{\text{m}}\sin\left(\omega_{\text{m}}t\right)+\ldots \right)\cdot \left[\Delta^{2}+\frac{V_{\text{AC}}^{2}}{2}\sin^{2}\left(\frac{\Delta \omega_{\text{e}}}{2}t\right)\right]$$

[10] $$=\frac{1}{2}C\prime \left[\Delta^{2}+\frac{V_{\text{AC}}^{2}}{4}\right]+\frac{1}{8}C\prime V_{\text{AC}}^{2}\cos\left(\Delta \omega_{\text{e}}t\right)$$

[11] $$+\frac{1}{2}{C^{\prime }}^{\prime }A_{\text{m}}\left[\Delta^{2}+\frac{V_{\text{AC}}^{2}}{4}\right]\sin\left(\omega_{\text{m}}t\right)+\frac{1}{16}{C^{\prime }}^{\prime }A_{\text{m}}V_{\text{AC}}^{2}\sin\left[\left(\omega_{\text{m}}\pm \Delta \omega_{\text{e}}\right)t\right].$$

In addition to a static force term identical to Equation 3, Equation 10 contains a term proportional to *C*′ at frequency 2ω_mod_ *=* Δω_e_. This force has been used for AM-based dielectric spectroscopy [63,69,74,84–88]. The second term, Equation 11, contains a force component at the mechanical drive frequency ω_m_ and at a sideband frequency ω_m_ ± 2ω_mod_. The latter one is independent of the local CPD, making it interesting for dielectric measurements. As the magnitude of this force component depends on *C*″, we can expect a superior lateral resolution through a reduction of long-range force contributions from tip cone and cantilever. As in the case of conventional EFM, signal-to-noise is greatly improved by choosing Δω_e_ such that one of the induced sidebands falls on one of the cantilever’s mechanical resonances. We call this method multifrequency heterodyne electrostatic force microscopy (MFH-EFM).

To calculate the second capacitance gradient, we need to calculate the electrostatic force from the detected amplitude signal, *A*_det_, taking into account the cantilever’s frequency-dependent spring constant or transfer function, *k*(ω):


[12] $$\frac{\partial^{2}C}{\partial z^{2}}={C^{\prime }}^{\prime }=\frac{16A_{\text{det}}\cdot k\left(\omega \right)}{A_{\text{m}}\cdot V_{\text{AC}}^{2}}.$$

Interestingly, the forces in Equation 11 only depend on the frequency difference, Δω_e_, of the electrical drive frequencies. Thus, the experiments can be performed at almost arbitrarily high AC frequencies. The lower limit for the frequency range is given by the second resonance of the cantilever. Towards higher frequencies, the impedance of the electrical connection will introduce a damping of the excitation signal that has to be considered in Equation 12. By using appropriate means of coupling the electrical excitation into the tip–sample gap, experiments at microwave or even at optical frequencies are possible. In our setup, the two excitation frequencies can be varied in frequency from ≈600 kHz up to at least 50 MHz, limited by the bandwidth of the LIA. To reach a nanoscale-sensitive measurement of the dielectric constant in media besides air, a detection at higher excitation frequencies in the megahertz regime is strictly necessary [59].

The indirect detection of local capacitance variations by means of an electrostatic force has the advantage that it does not require additional devices for the measurement except for the LIA similar to that in the work of Gramse and colleagues [56]. Nevertheless, quantifying the total tip–sample capacitance will require varying the distance, for example, by force–distance spectroscopy.

## Methods

### Multifrequency heterodyne electrostatic force microscopy to measure the second capacitive gradient *C*″

**Figure 1:**
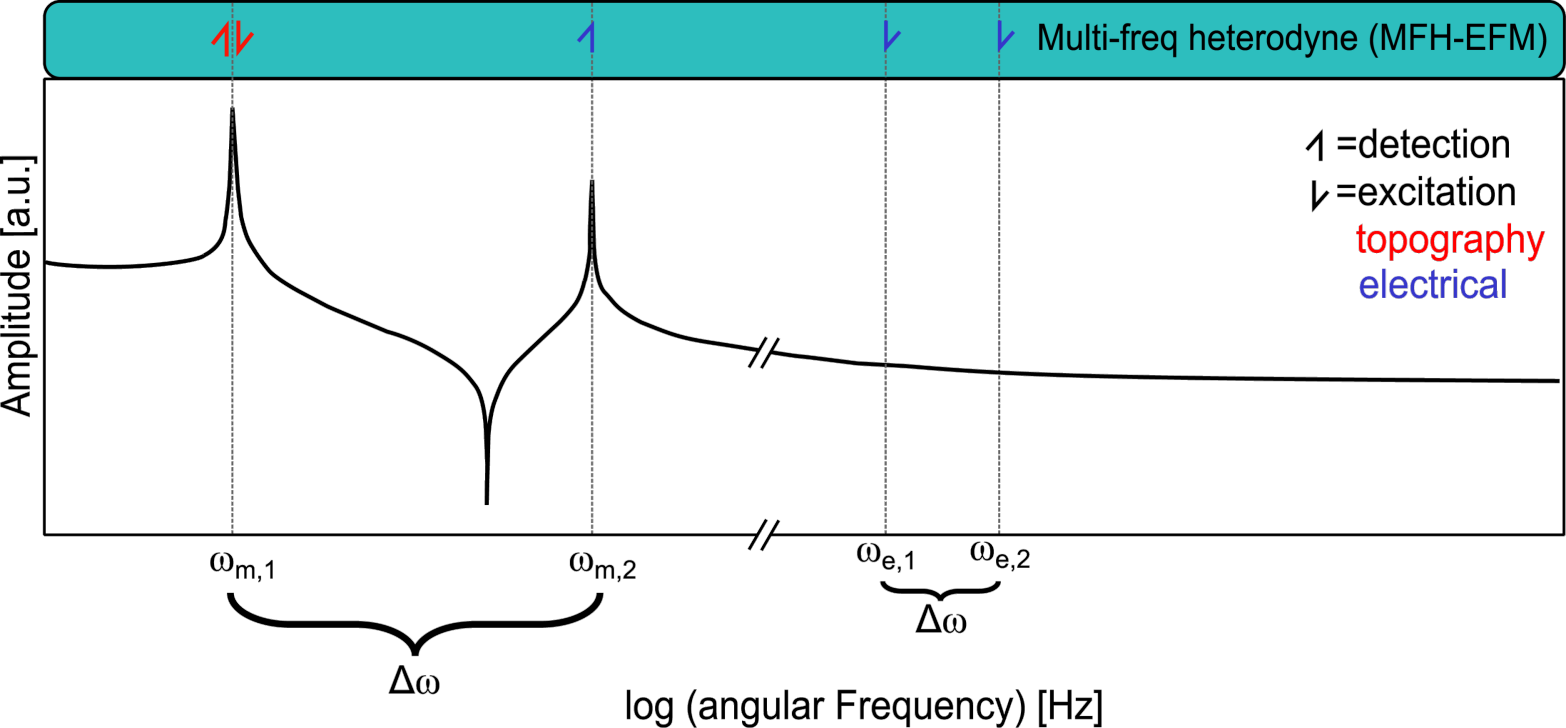
Schematic illustration of the excitation and detection frequencies in MFH-EFM. The lower part shows the transfer function of the cantilever, where the amplitude is plotted as function of the logarithmic angular frequency. The upper part shows the excitation frequencies (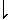) and the detection frequencies (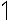) of the applied frequencies. The red arrow corresponds to topography, and the blue arrow corresponds to the electrical signal. The representation of Figure 1 was inspired by [18,26]. A comparison of heterodyne Kelvin probe force microscopy (H-KPFM) and MFH-EFM can be found in Figure S1, Supporting Information File 1.

We perform MFH-EFM using a conductive AFM cantilever in tapping mode with a mechanical drive near the fundamental cantilever eigenmode ω_m,1_ with a mechanical amplitude *A*_m_. Additionally, we apply two high-frequency electrical excitations of identical magnitude (*V*_AC,1_ *= V*_AC,2_) at the frequencies ω_e,1_ and ω_e,2_ (see Equation 7). A schematic of the excitation frequencies is shown in Figure 1.

We select the excitation frequencies at the *n*-th and the (*n* + 1)-th multiple of the frequency gap Δω = (ω_m,2_ − ω_m,1_) (see Figure 1). Note that the use of integer multiples is a technical limitation coming from our LIA. In principle, any combination of frequencies with Δω = (ω_m,2_ − ω_m,1_) would work. We then use lock-in detection to measure the induced mechanical excitation exactly at the second mechanical resonance of the cantilever (ω_m,2_).

### Single-frequency electrostatic force microscopy to measure the first capacitive gradient *C*′

To obtain a quantitative comparison of the signal contributions to the signals based on the first and the second capacitance gradient, we performed single-frequency excitation EFM (SF-EFM) measurements as comparison to the multifrequency approach described above. In the fixed-frequency configuration, we use lock-in amplification to detect the second harmonic force component at 2ω_e_ induced by a single-frequency (ω_E_) stimulus (see Equation 5).

To enhance the signal, we select ω_E_ such that 2ω_E_ coincides with the second resonance of the cantilever (2ω_E_ = ω_m,2_). We connect the numerical value of the capacitance gradient to the detected amplitude using the cantilever’s frequency-dependent transfer function or spring constant *k*(ω) by


[13] $$\frac{\partial C}{\partial z}=C\prime =\frac{4A_{\text{det}}\cdot k\left(\omega \right)}{V_{\text{AC}}^{2}}.$$

For the variable-frequency detection of *C*′, we apply two AC voltages of the same magnitude (*V*_AC,1_ = *V*_AC,2_) at frequencies *n* and (*n* + 1) times the second resonance frequency ω_m,2_. According to Equation 10, this will excite an oscillation at ω_m,2_ with an amplitude proportional to *C*′.

### Silicon microcapacitors

**Figure 2:**
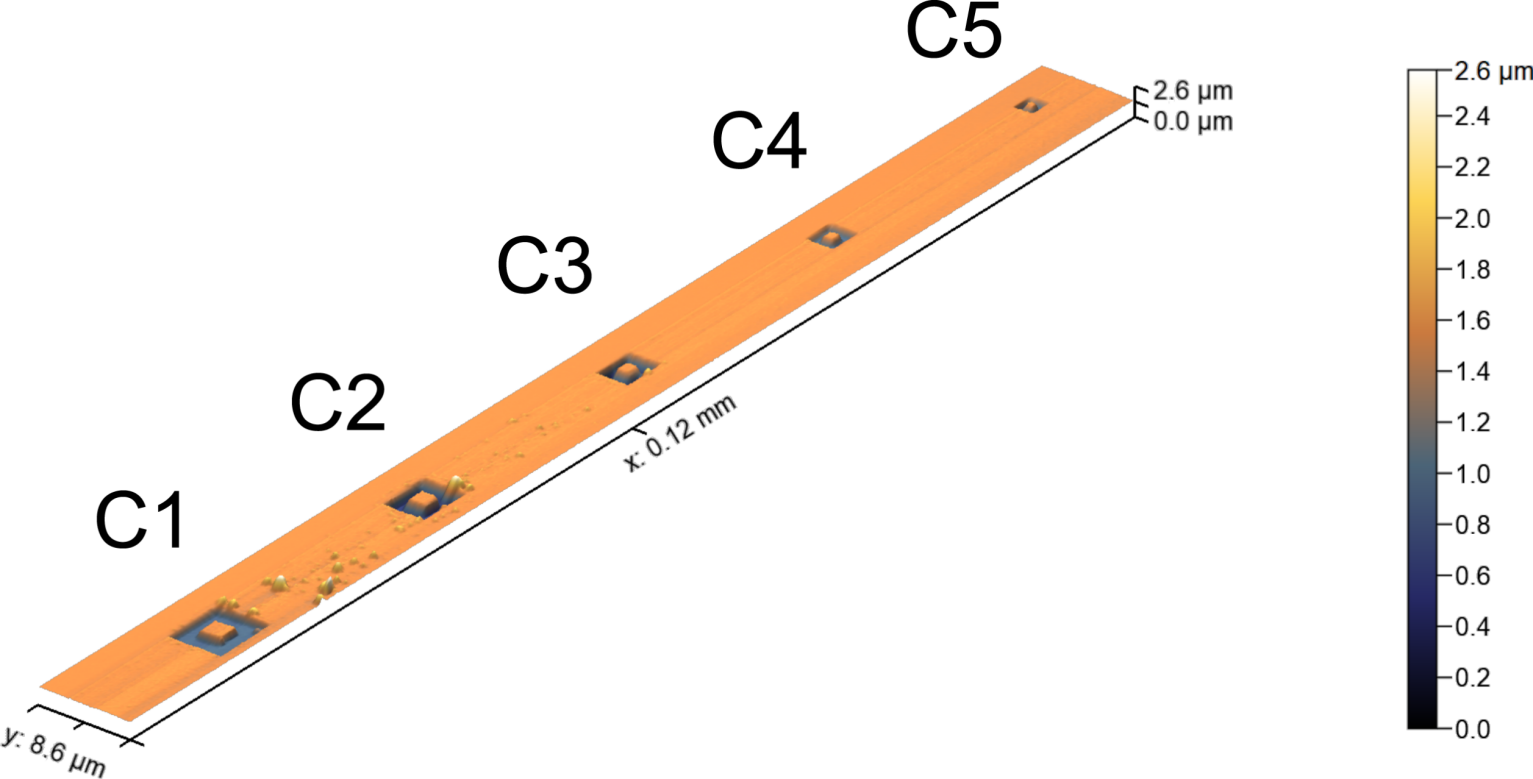
Topography of the five different capacitors C1 to C5 that were produced to have specific capacitors with known capacitance. Raw *C*″ pictures measured in MFH-EFM mode are shown in Figure S2, Supporting Information File 1. The topography measurement was conducted with a MikroMasch HQ:NSC18/Pt cantilever and analyzed with Gwyddion 2.61.

To generate structures with a defined dielectric response, we prepared a series of microcapacitors. We used these structures to compare the *C*′ and *C*″ distance dependencies from several force–distance curves with model calculations using tip–sample models from the literature, as well as for dielectric nanospectroscopy experiments. The microcapacitors were produced by focused ion beam (FIB) milling on a silicon wafer with a 300 nm layer of SiO_2_ and a 14 nm sputtered layer of Pt on it (Figure 2).

## Results and Discussion

To investigate whether the *C*″-sensitive detection leads to an improved spatial resolution of MFH-EFM as compared to conventional methods, we calculate the distance dependence of the first- and second-order capacitance gradients in an ideal cantilever. We compare our calculations to experimentally obtained force–distance curves. We then show the first practical examples of high-frequency capacitive spectra obtained by this method on etched SiO_2_ microcapacitors, along with high-resolution high-frequency capacitance images obtained over self-assembled molecular F14H20.

### Tip–sample capacitance

**Figure 3:**
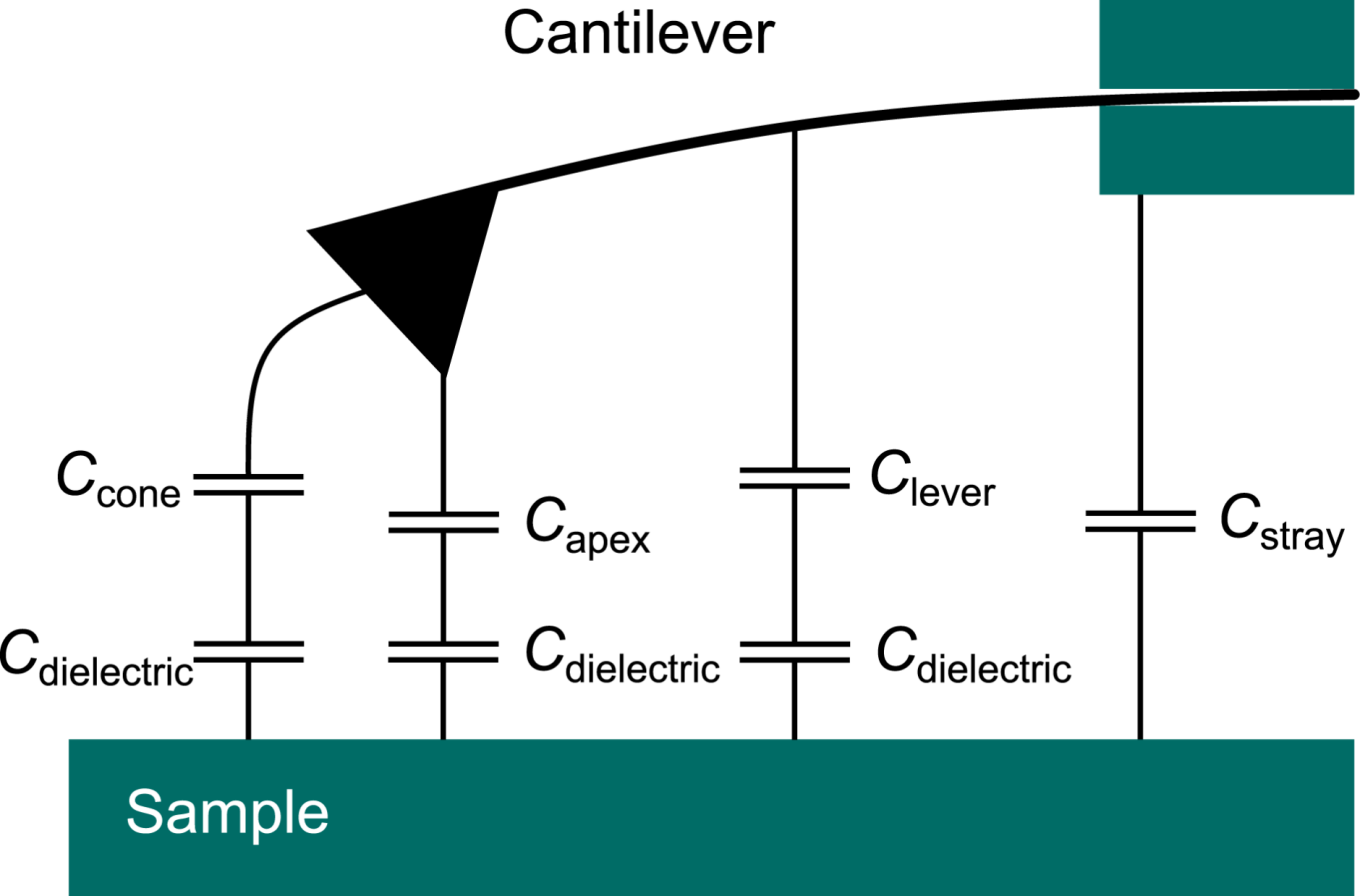
Schematic illustration of tip apex, tip cone, lever, and stray capacitances. The contribution of the tip apex contains the most localized part of the overall capacitance signal. The mesoscopic tip cone and the macroscopic cantilever, in contrast, contribute to the long-range stray capacitance, effectively delocalizing the signal.

The total capacitance between sample and cantilever consists of contributions from tip apex, tip cone, lever, and some additional stray capacitance caused by the signal cables in the AFM head (Figure 3). In the case of a dielectric sample, the tip-, apex- and lever-surface capacitors are connected in series with capacitors formed by the sample dielectric layer. The exact configuration for these capacitors depends strongly on the local electric field distribution around tip apex, tip cone, and cantilever. Whereas the apex capacitance contains the desired local information, the stray capacitance from cone, lever, and cables produces a background signal that effectively reduces the lateral resolution of the local capacitance measurement. Practically, these signal contributions can be discerned by their respective distance dependence.

To further investigate this distance dependence, we compare experimental force–distance spectra to analytical and numeric models from the literature. In particular, we combine the models for the apex contribution of Hudlet et al. [89] with the cone and lever contributions from Colchero and colleagues [90–91]. The full equations for the force together with the resulting capacitance used here are given in the Appendix section (see Equations Equation 14–Equation 15 and Figure 10 below).

**Figure 4:**
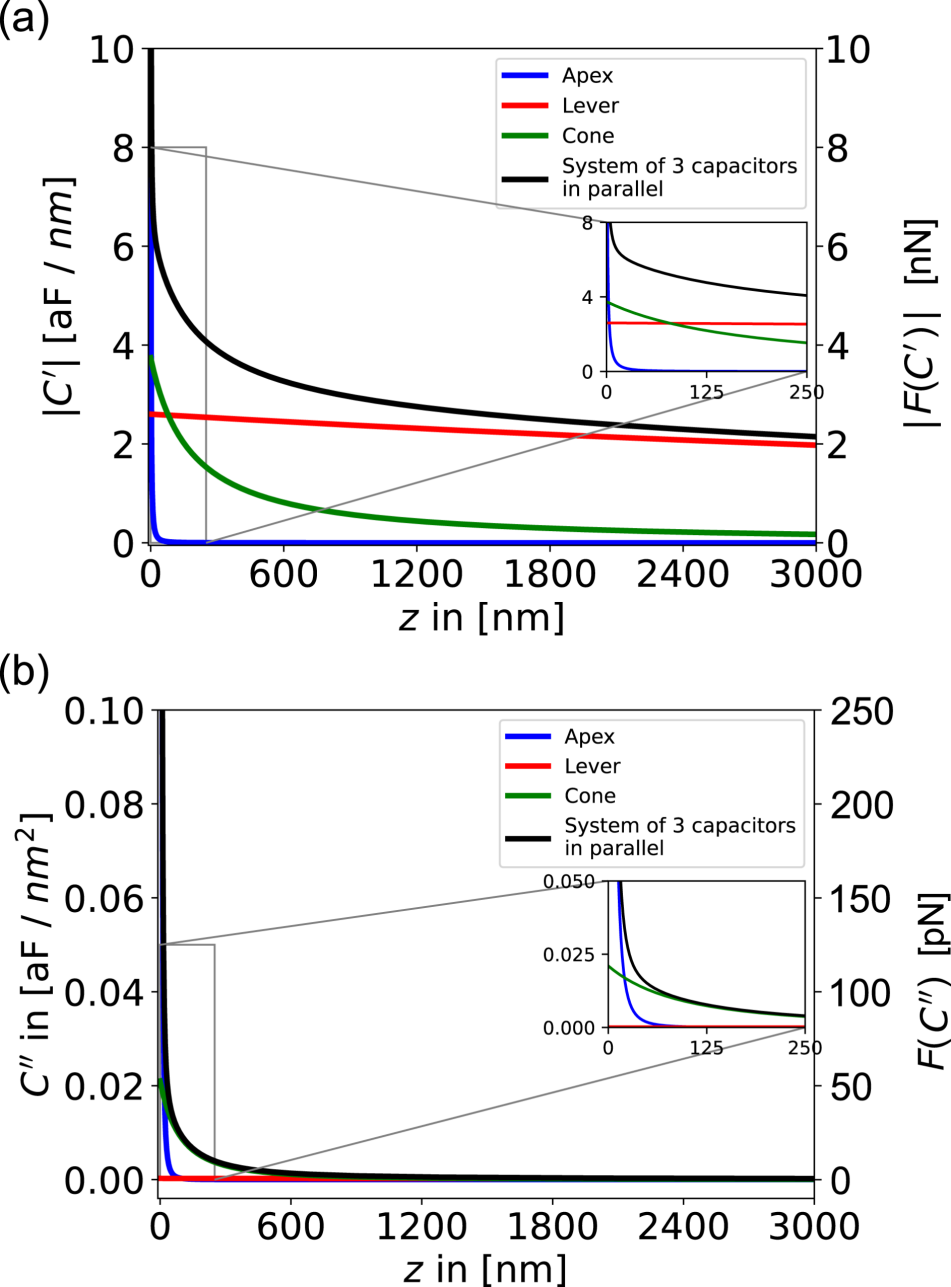
Contributions of the respective components to the (a) first numeric derivative *C*′ and (b) second numeric derivative *C*″ of the capacitance as functions of the distance *z* between tip and sample. Additionally, the respective forces (a) *F*(*C*′) and (b) *F*(*C*″) were plotted as functions of *z*. A NuNano SPARK 70 Pt cantilever (*w* = 30 μm, *l* = 225 μm, α = 11°, *h* = 12 μm, θ = 25°, *r* = 18 nm, and δ = 3.7·10^−7^) was used for the calculations with an mechanical amplitude of *A*_m_ = 10 nm, an excitation voltage of *V*_AC_ = 2 V, and a total amount of calculated points of 100,000. The blue line marks the apex, the green line the cone, the red line the lever, and the black line marks the entire system of the three components in parallel.

In Figure 4, we compare the respective contributions to the first and second capacitance gradients together with the corresponding electrostatic forces during a typical AFM experiment as functions of tip–sample distance *z*. For the force calculations, we used Equation 12 together with the parameters of a regular EFM cantilever (NuNano SPARK 70 Pt) and an electrical drive of *V* = 2 V and a mechanical amplitude of *A*_m_ = 10 nm. Comparing the graphs, we can immediately see that the total *C*′ signal retains a significant long-range contribution even at a tip–sample separation of 3000 nm (Figure 4a). In contrast, the *C*″ signal drops more rapidly over a short distance *z* (Figure 4b), indicating a reduced influence of long-range contributions to the force signals.

A measure of how much the signal is disturbed by non-local long-range contributions is the apex contribution to the total signal at a given distance *z*. At a typical tip–sample separation of 10 nm, the apex signal makes up more than 82% of the complete *C*″ signal, while the apex contribution to the first capacitance gradient only makes up less than 10% of the total *C*′ signal. In closer proximity of 1 nm distance to the sample, the apex contribution to the *C*″ signal increases to 99.8%, whereas the *C*′ signal still contains a significant amount of non-local signal contributions with 62% apex vs 38% cone and lever signal. Another way to quantify the “locality” of a force signal is to investigate the tip–sample separation at which the tip apex contribution surpasses the lever-plus-cone contributions within Figure 4. This is true in Figure 4a for distances smaller than ≈3 nm, while in Figure 4b, this is the case even for distances smaller than ≈20 nm. Comparing the absolute values of the forces, however, we see that MFH-EFM yields much weaker forces: At a tip–sample distance of 10 nm, the AM-based operation leads to a force of *F*_ES_(*C*^′^) = 6.7 nN, as compared to *F*_ES_(*C*″) = 280 pN for MFH-EFM. Hence, the resulting electrostatic force and, thereby, the expected force is by more than a factor of 24 lower for MFH-EFM. Thus, the improved lateral resolution comes at the price of a reduced signal-to-noise ratio.

**Figure 5:**
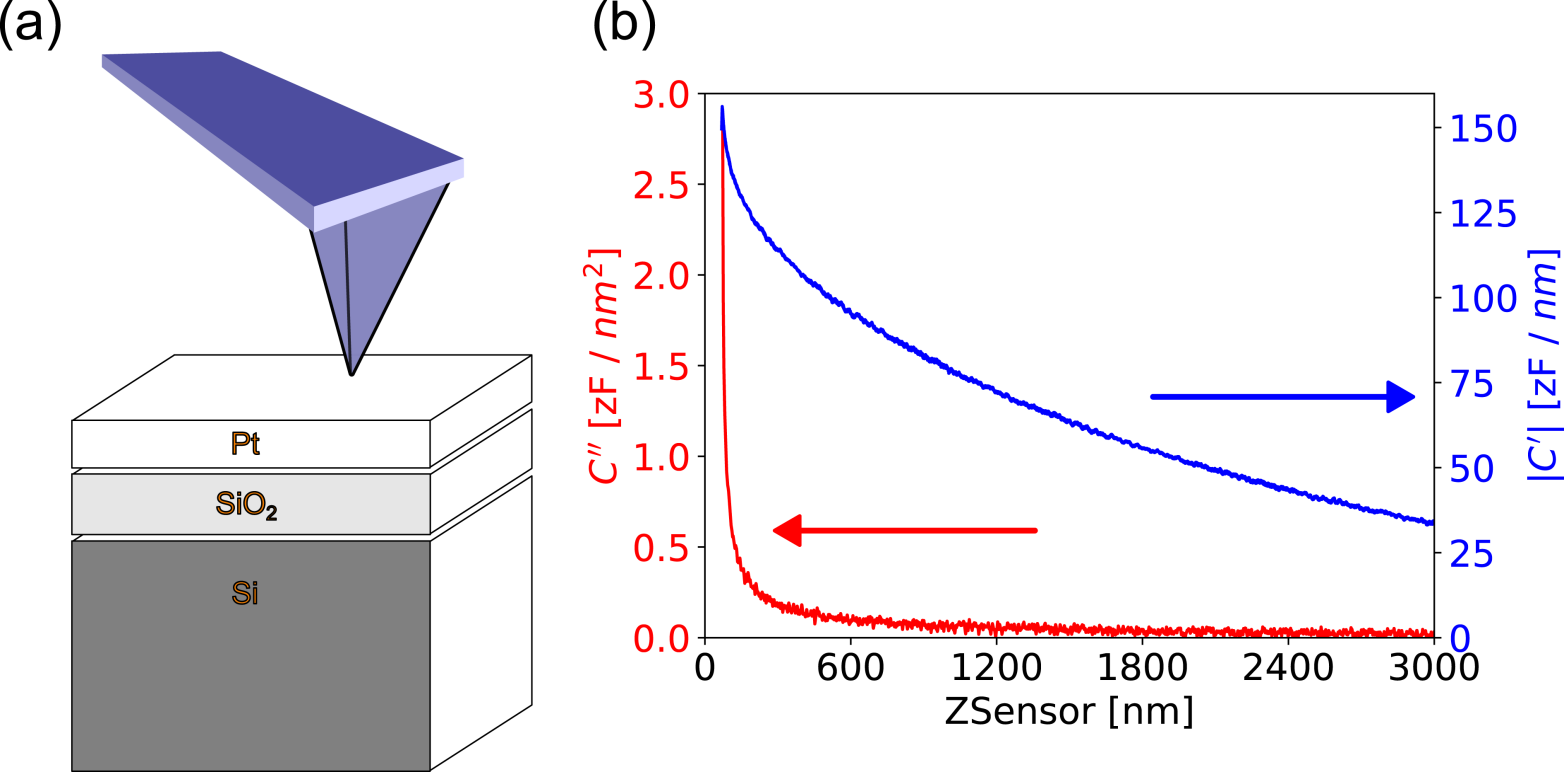
Comparison of the *C*″ and the *C*′ single force curves (b) of a microcapacitor (a) while doing MFH-EFM (see Equation 11 and Equation 12) and compared with the detection of 2ω (see Equation 5 and Equation 13). The measurement was conducted with a NuNano SPARK 70 Pt cantilever.

To reproduce these findings experimentally, we performed force–distance spectroscopy on the etched microcapacitors shown in Figure 2. The resulting curves of the *C*′ and *C*″ signals qualitatively reproduced the simulation results (Figure 5). Whereas the *C*″ signal only emerged from the noise at distances of less than 500 nm, the *C*′ signal shows a monotonic decrease over the full 3 μm of vertical travel. Compared to the simulations, the experimental *C*′ signal shows a slower decrease, indicating a stronger influence from the tip cone. The direct comparison of the model and the data of the second and first capacitance gradients can be found in Figure S16 and Figure S17, Supporting Information File 1, respectively. These results clearly show that the MFH-EFM method produced an electrostatic force signal that is highly local with suppressed stray contributions from cone and lever.

### Dielectric nanospectroscopy

**Figure 6:**
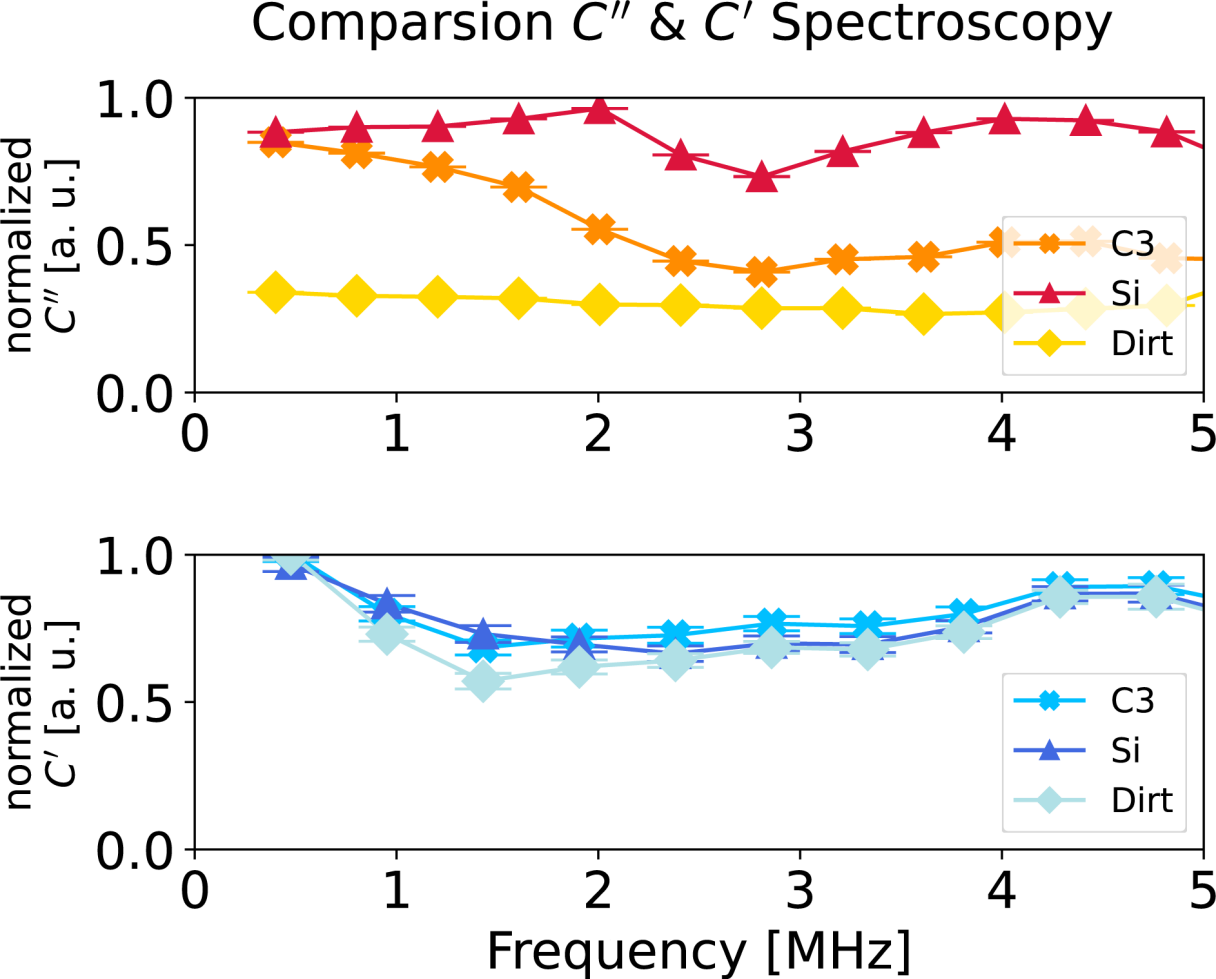
Comparison of the normalized *C*″ (red colors on top) and normalized *C*′ (blue colors at the bottom) frequency sweep on one of the capacitors (C3, see Figure 2, cross symbols), on milled silicon (Si, triangle symbols), and on a particle of unknown origin (Dirt, square symbols). This experiment was conducted with a MikroMasch HQ:NSC18/Pt cantilever. The non-normalized data, as well as normalized data over a wider frequency range, can be viewed in Figures S3–S6 and S9–S12, Supporting Information File 1.

The advantage of the multifrequency excitation approach of MFH-EFM is that we can choose arbitrary frequencies above the detection frequency for the electrostatic excitation. As the tip–sample capacitance is influenced by the dielectric properties of the material in the tip–sample gap (see Figure 3), the frequency-dependent electrostatic force represents the local dielectric function. To demonstrate the feasibility of dielectric nanospectroscopy, we performed MFH-EFM frequency spectroscopy at three different locations on the microcapacitor sample. The first spectrum was recorded on one of the microcapacitors (*C*3, see Figure 2). Then, we measured in one of the FIB-etched trenches around the capacitors. Here, we assume that the bare silicon surface is covered by a thin native oxide layer (Si). Last, we measured on a particle of unknown origin (Dirt, visible in Figure 2). The frequency sweeps were performed by keeping the tip position and amplitude fixed, varying the two heterodyne excitation frequencies while keeping their separation fixed, and recording the resulting excitation amplitude at the second mechanical resonance. All spectra were normalized against a reference spectrum recorded on the bare substrate far away from the capacitors to compensate any frequency response arising from the stray capacitance in the signal paths and cantilever. The electrostatic signal of the capacitor C3 showed a drop at around 2 MHz in Figure 6. When considering the capacitance of C3 of 183 ± 1 aF and the drop-off frequency ω_d_ of the capacitance at 1.7 MHz, we can calculate the resistance *R* via the RC time (*RC* = 1/ω_d_) as *R* ≈ 3200 MΩ. This value is much smaller compared to the calculated value of the resistance of SiO_2_, which is 25·10^21^ Ω, taking into account the electrical resistivity of silicon dioxide of 
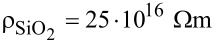
 [92] and a thickness of the SiO_2_ layer of 300 nm on an area of 9 μm^2^. The observed discrepancy may be attributed to the increased conductivity of the microcapacitors, which is a result of the incorporation of Ga^+^ ions into the SiO_2_ layer.

The *C*″ signal of the bare Si was stable over the whole range of excitation frequencies and only dropped at a much higher frequency around 24 MHz (see Figure S5, Supporting Information File 1). The dielectric response of the undefined particle was significantly lower compared to the response of the capacitor structures. In the frequency response, we found little to no signal response, even at low excitation frequency. A rise of the signal at around 6 MHz could be observed in all the *C*″ signals at that frequency (see Figure S3, Supporting Information File 1), which we attribute to a capacitive singularity in the electrical connection to the sample. We observed a similar behavior in the frequency range between 5 and 10 MHz and around 17 MHz. We want to point out that we used standard AFM equipment with no special means to control the impedance of the electrical connections. To obtain more trustworthy data in the frequency range above 5 MHz, specialized sample and cantilever holders with coaxial electric connections will be required.

To compare these results with the conventional AM-based EFM approach, we repeated the spectroscopy experiments for the *C*′ signal based on the second term in Equation 10 (Figure 6, non-normalized data in Figures S9–S11, Supporting Information File 1). In comparison to the MFH-EFM data, the *C*′ frequency sweep looked very similar on the different structures. We think that this reduction in contrast is caused by the stronger influence of the long-range interactions from tip cone and cantilever in the *C*′ signal. Thus, the overall impact of the local surface dielectric properties under the tip apex is reduced as compared to the impact of the dielectric properties probed by tip cone and cantilever (see Figure 3).

### Imaging C′ versus C″

**Figure 7:**
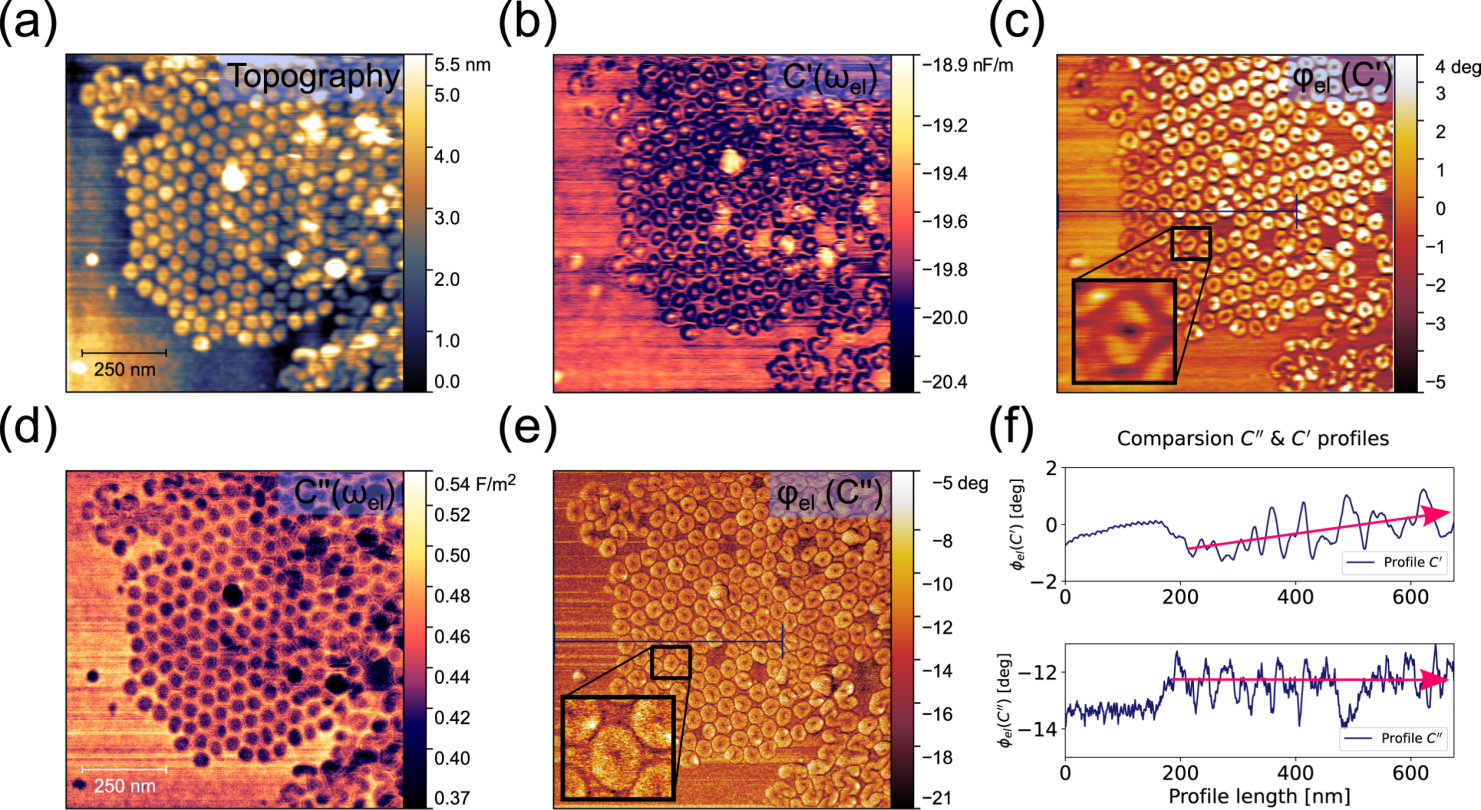
MFH-EFM images taken on F14H20. (a) Topography image. (b) *C*′ image detected at ω_m,2_ under excitation at 235.579 kHz. (c) Electric phase φ_el_ of the *C*′ signal detected at ω_m,2_ under excitation at 235.579 kHz. (d) *C*″ image detected at ω_m,2_ under excitation at frequencies of 1.59 and 1.98 MHz. (e) Electric phase φ_el_ of the *C*″ signal detected at ω_m,2_ under excitation at frequencies of 1.59 and 1.98 MHz. (f) Profiles of the phase images shown in (c) and (e) with 128 pixels width and the same resolution. The full picture can be found in Figure S15, Supporting Information File 1. The measurements were conducted with a MikroMasch HQ:NSC18/Pt cantilever.

To demonstrate the capabilities of MFH-EFM as an imaging method, we performed experiments on self-assembled nanostructures consisting of the amphiphilic molecule F14H20 (Figure 7). F14H20 exhibits a strong dipole moment of 3.1 D oriented along the chains consisting of fluorinated and hydrogenated parts [93], leading to a strong nanoscale contrast in the dielectric signal.

On the silicon substrate, F14H20 formed groups of spherical particles with a diameter of 40 ± 5 nm (Figure 7a) [94–95]. Simultaneously with the topography, we recorded the *C*″ amplitude and phase at electrical excitation frequencies of 1.59 and 1.98 MHz. In the dielectric spectroscopy images, we see a sharp contrast between the F14H20 particles and the silicon substrate, both in *C*′ (Figure 7b,c) and *C*″ (Figure 7d,e). Within all images, the particles exhibit a lower amplitude signal than the surroundings [95]. The latter is formed by a thin fluoroalkane layer with molecules lying along the sample surface [95]. The contrast within the particles correlates with variations of dielectric permittivity, and the latter is related to averaged dipole values [95]. Similar work [96] indicated that the response increases with an increase of sample permittivity [95–96]. We measured a CPD difference between Si and F14H20 of −0.72 ± 0.08 V (see Figure S15, Supporting Information File 1), which is close to the literature value of −0.8 V [97].

Interestingly, the image of the *C*′ signal (Figure 7c,e) showed a more blurry structure (compare the insets in Figure 7b,c). Another effect that can be observed in the *C*′ phase images is that the individual contrast on the particles changes when going towards the center of the particle agglomerate (upper graph in Figure 7f). While there is only a very shallow contrast for the first two to five particles, both the contrast and the baseline signals increased towards the center of the agglomerate. In the *C*″ images, however, the dielectric contrast remained the same across the particle agglomerate, demonstrating once more that MFH-EFM provides more local information and is less affected by long-range electrostatic effects.

## Conclusion

We have presented a novel method for high-resolution nanoscale capacitance characterization based on multifrequency electrostatic force microscopy, complementing established methods in the field. The key advantage of the multifrequency approach of MFH-EFM is that it allows for measurements of higher-order tip–sample capacitance gradients at almost arbitrary frequencies above the second cantilever resonance, enabling the measurement of the local dielectric function over a wide range of frequencies. In comparison to many existing SCM operation modes, MFH-EFM leads to a significant reduction of signal background, which results in higher locality of the measurements with less cross talk. This is due to the fact that the second capacitance gradient is less affected by long-range interactions, such as those from tip cone and lever. We demonstrate the reliable operation using standard AFM equipment together with an external LIA up to a frequency of 5 MHz. At higher frequencies (up to 50 MHz in our case), the signals were dominated by impedance effects from the signal connections. Thus, to move towards reliable measurements at higher frequencies, specialized high-frequency equipment with coaxial signal connections will be required.

Our analytical simulations of the distance-dependent tip–sample capacitance showed that current models are not able to fully simulate the experimental data. Thus, to enable quantitative measurements of the tip–sample capacitance, further measures such as improved tip–sample models or full numerical simulations will be required. Here, the suppression of long-range electrostatic interactions in MFH-EFM could simplify the simulations. Thus, MFH-EFM could further improve quantitative studies on dielectric effects in nanoscale systems across materials science, biology, and nanotechnology.

## Experimental

### Polymer blend samples

We used F14H20 samples that we bought from SPM Labs LLC, Tempe, AZ, USA.

### Microcapacitors

Si wafers “CZ” were bought from “Si-Mat” with a diameter of 150 mm, ⟨100⟩ surface orientation, a thickness of 675 ± 20 μm, a resistivity of 1.5–4.0 Ωcm, and with p-type doping with B atoms. These wafers were thermally oxidized with 300 nm SiO_2_. A compact coating unit 010/LV with the sputter head SP010 was used to sputter 14 nm of Pt on top of the wafer. The microcapacitors were then milled out of the surface using a FEI Nova600 Nanolab FIB apparatus with a dual Ga^+^ ion beam.

### Multifrequency heterodyne electrostatic force microscopy

MFH-EFM was measured on an Oxford Instruments/Asylum Research MFP-3D Infinity AFM in a nitrogen glovebox (level of humidity below 0.3%, level of oxygen below 0.1%). The typical resonance frequency of the Pt/Ir-coated conductive cantilevers (NuNano SPARK-150Pt and MikroMasch HQ:NSC18/Pt) was ≈75 kHz; the levers had a spring constant of 2–3 N·m^−1^, a tip radius of 18 nm, and a tip height of 10–18 μm. The topography feedback measurements were performed with amplitude modulation on the first eigenmode ω_m,1_, and the oscillation amplitude was kept to 70–90 nm for all measurements. The force spectroscopy measurements were done with a *z* rate of 0.2 Hz and a force distance of 8 μm for all samples.

**Figure 8:**
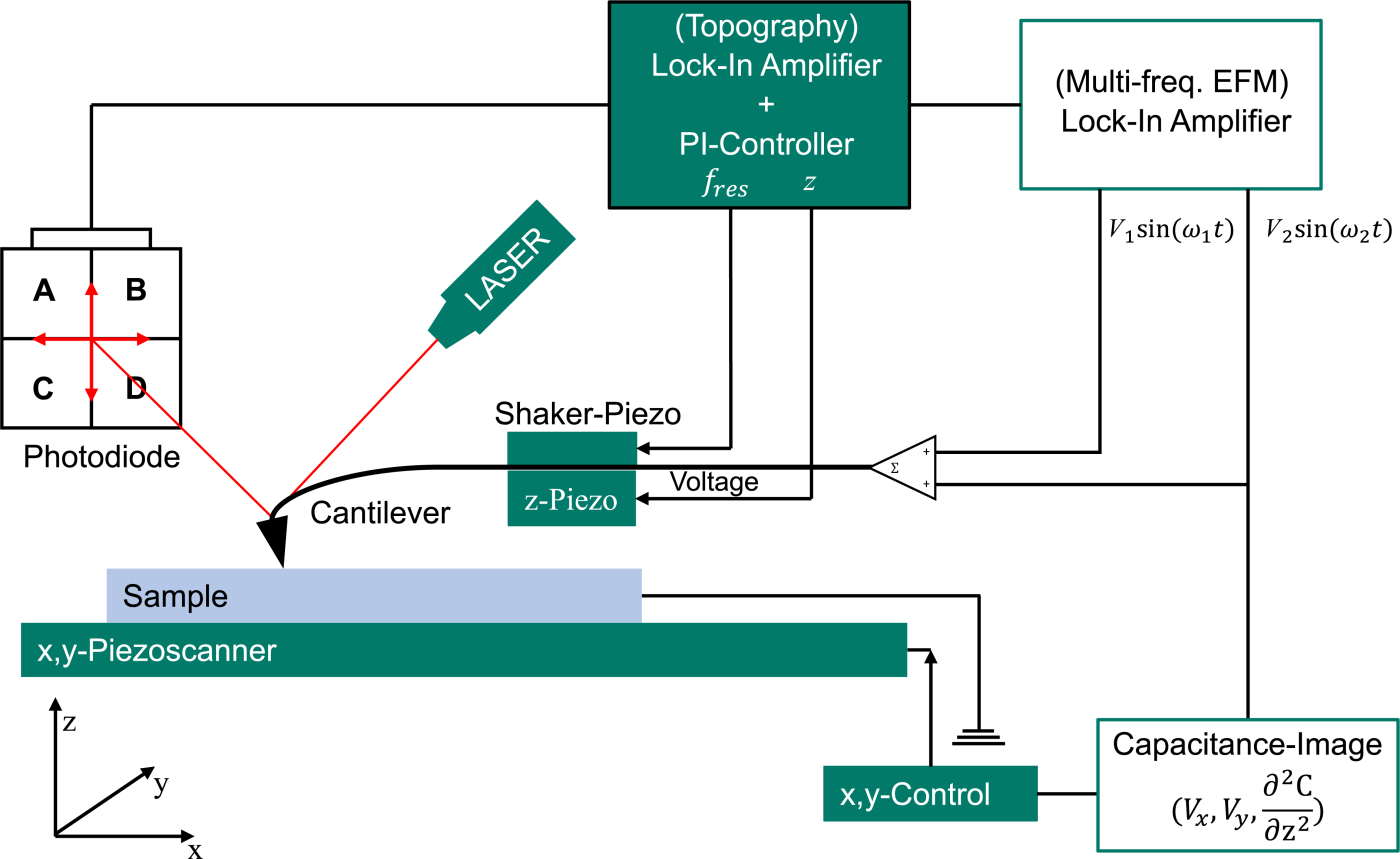
Schematic setup of the MFH-EFM apparatus. Additionally to a regular AFM, two different voltages with different frequencies are applied to the cantilever.

We used a Zurich Instruments HF2 LIA for all experiments. The electric drive amplitude of the *V*_AC,1_ = *V*_AC,2_ signal varied between 3 and 5 V, depending on the obtained signal from the sample. We grounded the sample via the sample holder with an external wire to ground level of the LIA. The *V*_AC_ was applied to the tip directly, while the AFM head connections were switched off. The setup of the AFM is shown in Figure 8. The electrical connection from the LIA to the cantilever with the two excitation voltages was realized by using a direct cable connection.

### Focused ion beam milling

FIB milling of the cantilever was conducted using a LEO Gemini instrument from Zeiss. It was used with an acceleration voltage of 3 kV.

## Appendix

### Equations to calculate the *C*″ and *C*′ signal from the voltages

Equation 16 shows a detailed expression of Equation 12. The detected amplitude from the LIA, *A*_det_, contains the voltage from the LIA (*V*_MFH−EFM_) and Ξ_amp,d2C_, the amplification factor of this voltage from the LIA in MFH-EFM mode. The frequency-dependent spring constant *k*(ω) in Equation 12 contains the inverse optical lever sensitivity (InvOLS) of the second harmonic (InvOLS_2_), the spring constant of the second resonance (*k*_2_), and the Q-factor shown in Equation 16. It is important to note that the InvOLS and the spring constant on the second resonance are not the same as measured on the first resonance by the method of Sader and colleagues [98]. It is rather necessary to calculate the properties of the cantilever for the respective eigenmodes [99].


[16] $$\frac{\partial^{2}C}{\partial z^{2}}(\omega )=C\prime \prime (\omega )=\frac{16\cdot V_{\text{MFH-SCM}}(\omega )\cdot InvOLS_{2}(\omega )\cdot k_{2}(\omega )}{A_{\text{m}}\cdot V_{\text{AC}}^{2}\cdot \Xi_{\text{amp,d2C}}\cdot Q}$$

Equation 17 shows a detailed expression of Equation 13. Again, the expression *A*_det_ contains the detected voltage from the LIA (*V*_SF−EFM_) and an amplification factor Ξ_amp,dC_ of the signal captured with the LIA in SF-EFM mode. The frequency-dependent spring constant *k*(ω) is the same as above and consists of InvOLS_2_, *k*_2_, and the Q-factor.


[17] $$\frac{\partial C}{\partial z}\left(\omega \right)=C\prime \left(\omega \right)=\frac{4\cdot V_{\text{SF-EFM}}\left(\omega \right)\cdot {\text{InvOLS}}_{2}\left(\omega \right)\cdot k_{2}\left(\omega \right)}{V_{\text{AC}}^{2}\cdot \Xi_{\text{amp},\text{dC}}\cdot Q}$$

### Full double excitation force equations

This section gives a full overview of the electric amplitude contributions at various frequencies while activating the MFH-EFM mode. For simplicity, we will use the following substitutions: 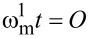, ω_e_*t* = *E*, ω_mod_*t* = *M*, *V*_CPD_ − *V*_DC_ = Δ, 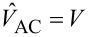, and 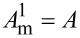. Table 1 shows the overview of the force components at various frequencies for the resulting static ω and 2ω force components acting on the cantilever.

**Table 1:** Overview of the components of the multifrequency electrostatic force microscopy.

Frequency	Amplitude

DC	1/2 *C*′ [Δ^2^ + *U*^2^/4]
2*M*	1/8 *C*′*U*^2^
*O*	1/2 *C*″*A*[Δ^2^ + *U*^2^/4]
*O* ± 2*M*	1/16 *C*″*AU*^2^
*E* ± *M*	1/2 *C*′*U*Δ
*O* ± (*E* ± *M*)	1/4 *C*″*AU*Δ
2*E*	1/8 *C*′*U*^2^
2(*M* ± *E*)	1/16 *C*′*U*^2^
*O* ± 2*E*	1/16 *C*″*AU*^2^
*O* ± 2(*E* ± *M*)	1/32 *C*″*AU*^2^

### Tip–sample capacitance model

**Figure 9:**
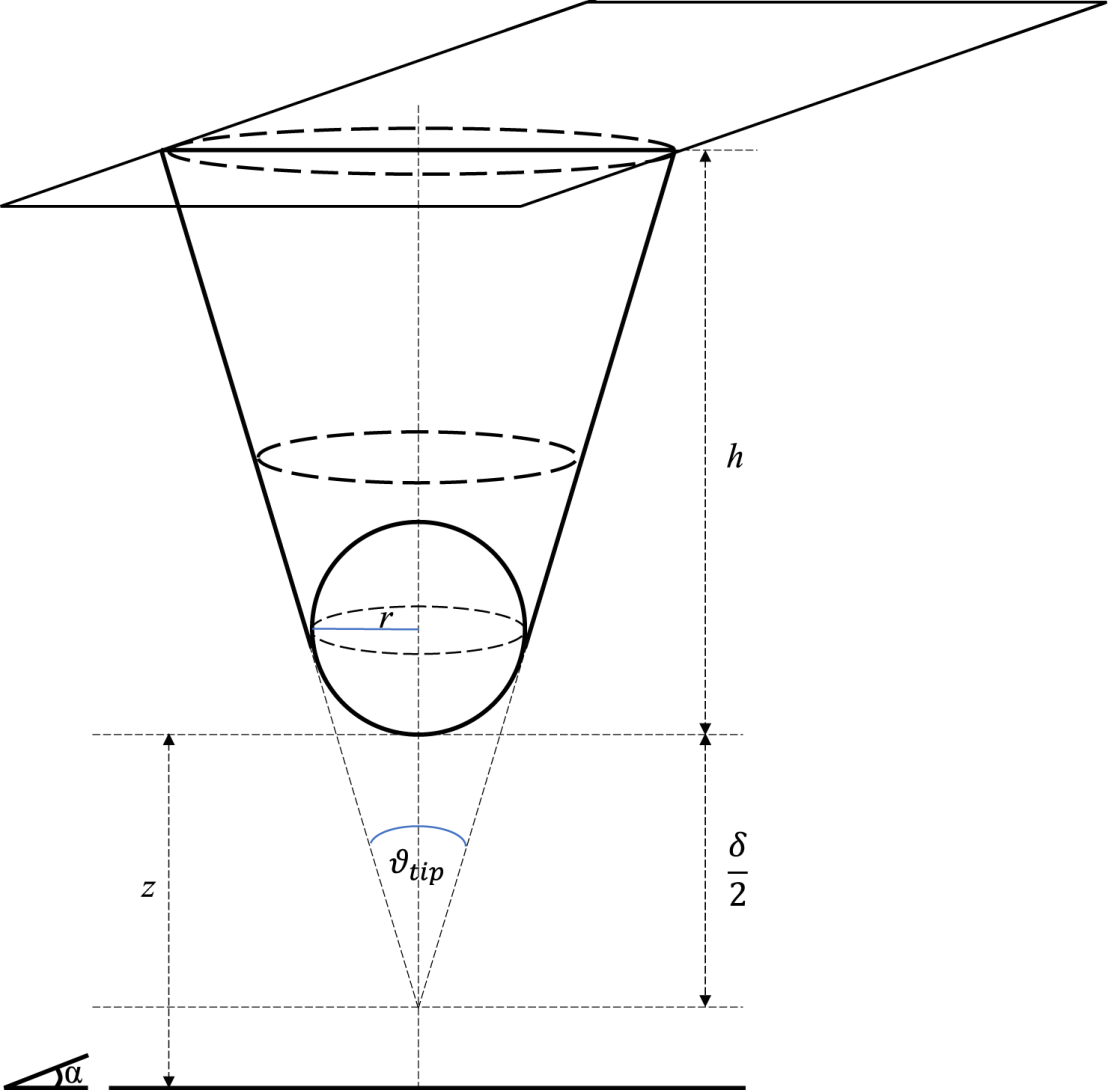
Sketch of the capacitance model of the truncated cone with spherical apex. Here *h* is the height of the tip, *r* is the radius of the sphere, ϑ_tip_ is the opening angle of the tip, δ is the truncated part of the cone, and *z* is the distance between sample and tip apex with respect to the surface normal of the sample. α is the angle between the surface and the lever of the cantilever.

We used the model of Hudlet et al. [89] for the tip apex and, in addition, used the sum of cone and lever distribution of Colchero and colleagues [90–91]. The cantilever can be modeled as a tilted plate capacitor with a truncated cone at the end of the cantilever and with a sharp round tip apex at the end of the tip cone. This is shown schematically in Figure 9.

In this case, the electrostatic force for the lever is given by Equation 14[90–91]:


[14] $$\begin{array}{c} F_{\text{lever}}\left(z\right)=\frac{2\tan^{2}\left(\frac{\alpha }{2}\right)}{\alpha^{2}}\varepsilon_{0}V_{\text{tip}-\text{sample}}^{2}\frac{lw}{h^{2}}\\ \cdot \frac{1}{\left[\left(1+\frac{z}{h}\right)\cdot \left(1+\frac{z+2l\tan\left(\frac{\alpha }{2}\right)}{h}\right)\right]}. \end{array}$$

Integration taking into account Equation 1 yields:


[18] $$\begin{array}{c} C_{\text{lever}}\left(z\right)=\frac{2\tan^{2}\left(\frac{\alpha }{2}\right))}{\alpha^{2}}\varepsilon_{0}V_{\text{tip}-\text{sample}}^{2}\frac{lw}{h^{2}}\\ \cdot \frac{h^{2}\cot\left(\frac{\alpha }{2}\right)\left\{\ln\left(h+z\right)-\ln\left[\cos\left(\frac{\alpha }{2}\right)\left(h+z\right)+2l\sin\left(\frac{\alpha }{2}\right)\right]\right\}}{2l}, \end{array}$$

where ε_0_ is the dielectric constant of the vacuum. The dimensions of the lever are given by its width *w*, its length *l*, and the height of the tip cone *h*. The lever is tilted by the angle α = ϑ_lever_.

The tip cone can be approximated by a truncated cone (Figure 9). The electrostatic force as a function of the distance between tip cone and sample is given by Equation 19[90–91]:


[19] $$\begin{array}{c} F_{\text{cone}}\left(z\right)=\frac{4\pi }{{\left(\pi -\vartheta_{\text{tip}}\right)}^{2}}\varepsilon_{0}V_{\text{tip}-\text{sample}}^{2}\\ \cdot \left[\ln\left(\frac{z-\frac{\delta }{2}+h}{z+\frac{\delta }{2}}\right)-\sin\left(\frac{\vartheta_{\text{tip}}}{2}\right)\frac{h-\delta }{z-\frac{\delta }{2}+h}\cdot \frac{z-\frac{\delta }{2}}{z+\frac{\delta }{2}}\right], \end{array}$$

with the open angle of the tip cone (ϑ_tip_) and the height of the truncated part of the cone (δ = *r*/tan^2^(ϑ_tip_/2)) [90–91]. Integration of this equation to obtain the capacitance yields


[20] $$\begin{array}{c} C_{\text{cone}}\left(z\right)=2\frac{4\pi \varepsilon_{0}}{{\left(\vartheta_{\text{tip}}-\pi \right)}^{2}}\\ \cdot \left\{\sin\left(\frac{\vartheta_{\text{tip}}}{2}\right)\left[h\ln\left(2f_{1}\right)-\delta \ln f_{2}\right]+f_{1}\ln\left(\frac{f_{2}}{2f_{1}}\right)+\left(\delta -h\right)\ln f_{2}\right\}, \end{array}$$

where 
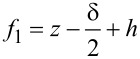
 and *f*_2_ = 2*z* + δ.

The tip apex is approximated as a sphere over an infinite surface (Figure 9). The corresponding electrostatic force between a tip apex and the surface is given by Equation 21[89]:


[21] $$F_{\text{apex}}\left(z\right)=\pi \varepsilon_{0}r^{2}V_{\text{tip}-\text{sample}}^{2}\left(\frac{1-\sin\left(\frac{\vartheta_{\text{tip}}}{2}\right)}{z\left\{z+r\left[1-\sin\left(\frac{\vartheta_{\text{tip}}}{2}\right)\right]\right\}}\right).$$

Hence, the capacitance is given by


[15] $$C_{\text{apex}}\left(z\right)=2\pi \varepsilon_{0}r\ln\left\{\frac{z+r\left[1-\sin\left(\frac{\vartheta_{\text{tip}}}{2}\right)\right]}{z}\right\}.$$

**Figure 10:**
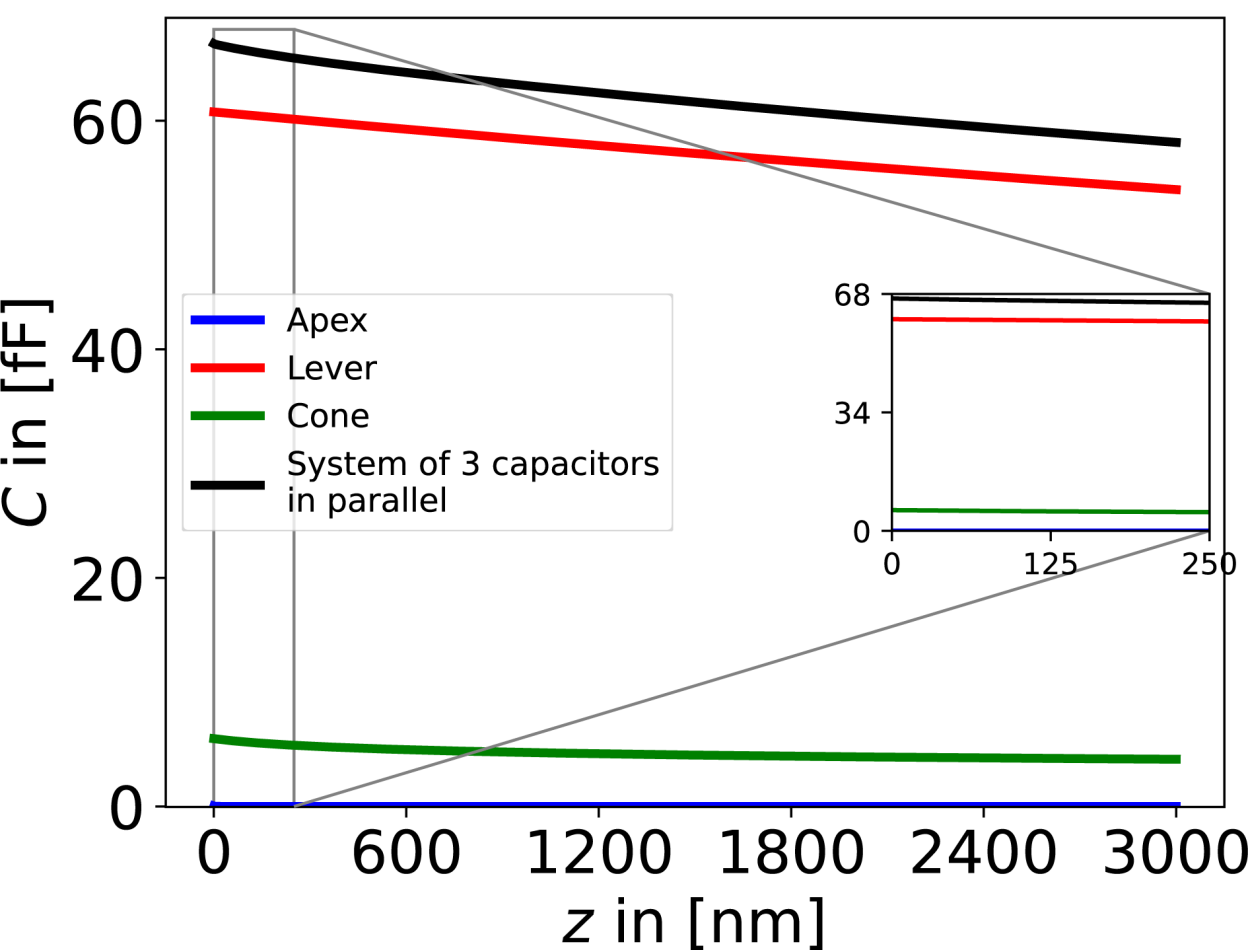
Contributions of the respective components to the numeric capacitance *C* as a function of the distance *z* between tip and sample. The properties of the NuNano SPARK 70 Pt cantilever (*w* = 30 μm, *l* = 225 μm, α = 11°, *h* = 12 μm, θ = 25°, *r* = 18 nm, and δ = 3.7·10^−7^) with a mechanical amplitude of *A*_m_ = 10 nm, an excitation voltage of *V*_AC_ = 2 V, and a total number of 100,000 calculated points, were used for the calculations. The blue line marks the apex, the green line the cone, the red line the lever, and the black line marks the entire system of the three components in parallel.

When the capacitance of the cantilever is plotted as function of the distance between the tip and the sample, *z*, Figure 10 is obtained. The parameters were taken from the website of the producer of the NuNano SPARK 70 Pt cantilever: *w* = 30 μm, *l* = 225 μm, α = 11°, *h* = 12 μm, ϑ_cone_ = 25°, *r* = 18 nm, and *V*_AC_ = 2 V.

In order to get the first, *C*′, and second capacitance gradient, *C*″, of the relevant parts of the cantilever, we used the onward and backward differentiation given in Equation 22 and the central differential quotient of the second order given in Equation 23, respectively. The step size was chosen to be 1·10^−10^ m with a total number of 1,000,000 steps. Models of the first and the second capacitance gradient can be found in Figure 4a and Figure 4b, respectively.


[22] $$f\prime \left(x\right)=\frac{f\left(x+h\right)-f\left(x-h\right)}{2h}$$

[23] $$f\prime \prime \left(x\right)=\frac{f\left(x+h\right)-2f\left(x\right)+f\left(x-h\right)}{h^{2}}$$

## Supporting Information

Supporting information features a comparison of the working principles of H-KPFM and MFH-EFM, all the raw and normalized data of the MFH-EFM frequency spectroscopy measurements, the full comparison of the MFH-EFM, SF-EFM, and H-KPFM images on the F14H20 structures, and finally a comparison of the model data and the measured data on the microcapacitors.

File 1 Additional experimental data.

## Data Availability

Data generated and analyzed during this study is available from the corresponding author upon reasonable request.
