# Supplementary material for: Nanoscale capacitance spectroscopy based on multifrequency electrostatic force microscopy
Source: Beilstein J Nanotechnol. 2025 May 8;16:637–51. doi: 10.3762/bjnano.16.49 (PMC12067100; doi:10.3762/bjnano.16.49)
Supplement: File 1 — Additional experimental data. [file Beilstein_J_Nanotechnol-16-637-s001.pdf]

BEILSTEIN JOURNAL OF NANOTECHNOLOGY

Supporting Information

for

Nanoscale capacitance spectroscopy based on multifrequency electrostatic force microscopy

Pascal N. Rohrbeck, Lukas D. Cavar, Franjo Weber, Peter G. Reichel, Mara Niebling and Stefan A. L. Weber

Beilstein J. Nanotechnol. **2025,** *16,* 637–651. doi:10.3762/bjnano.16.49

Additional experimental data

License and Terms: This is a supporting information file under the terms of the Creative Commons Attribution License (https://creativecommons.org/licenses/by/4.0). Please note that the reuse, redistribution and reproduction in particular requires that the author(s) and source are credited and that individual graphics may be subject to special legal provisions.
The license is subject to the *Beilstein Journal of Nanotechnology* terms and conditions: (https://www.beilstein-journals.org/bjnano/terms)

This Supporting Information features a comparison of the working principles of heterodyne Kelvin probe force microscopy (H-KPFM) and multi-frequency heterodyne electrostatic force microscopy (MFH-EFM), all the raw and normalized data of the MFH-EFM frequency spectroscopy, the full comparison of the MFH-EFM, SF-EFM, and H-KPFM images on the perfluoroalkyl-alkane $F(CF_2)_{14}(CH_2)_{20}H$ (F14H20) structures, and finally a comparison of the model data and the measured data on the microcapacitors.

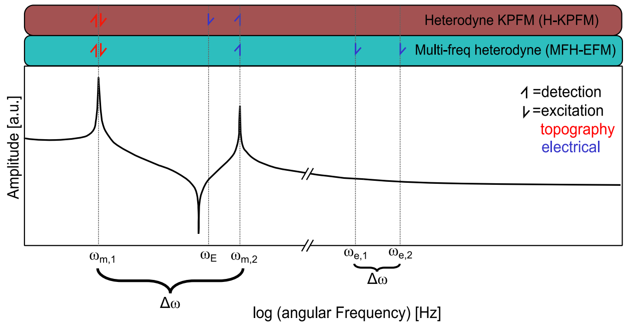

Figure S1: Schematic comparison of the excitation and detection frequencies in H-KPFM and MFH-EFM. The lower part shows the transfer function of the cantilever, where the amplitude is plotted vs the logarithmic angular frequency. The upper part shows the excitation frequencies (⇂) and the detection frequencies (↿) of the applied frequencies. The red arrow corresponds to topography- and the blue arrow to the electrical signal. Representation of Figure S1 was inspired by [1,2].

Table S1: Known capacitance methods from the literature compared to our new MFH-EFM method.

Reference in the Paper, DOI	Force detection method	Detecting second capacity gradient	High Freq. Sweep possible	Remarks
Jaensch2006 [3], 10.1016/j.physb.2005.12.227	NO	NO	NO	
Tran2002 [4], 10.1109/CCECE.2002.1015268	NO	NO	NO	dC/dV
				Continued on next page

Table S1 – continued from previous page

**Reference in the Paper, DOI**	**Force detection method**	**Detecting second capacity gradient**	**High Freq. Sweep possible**	**Remarks**
Raineri2001 [5], 10.4028/www.scientific.net/SSP.78-79.425	NO	NO	NO	dC/dV
Barrett1991 [6], 10.1063/1.349388	NO	NO	NO	
Yamamoto1996 [7], 10.1143/JJAP.35.3793	NO	NO	NO	dC/dV
Goto1997a [8], 10.1063/1.1147749	NO	NO	NO	dC/dV
Goto1997 [9], 10.1117/12.271216	NO	NO	NO	C
Fumagalli2006 [10], 10.1088/0957-4484/17/18/009	NO	NO	NO	
Fumagalli2007 [11], 10.1063/1.2821119	NO	NO	NO	
Gomila2008 [12], 10.1063/1.2957069	NO	NO	NO	
Fumagalli2009 [13], 10.1021/nl803851u	NO	NO	NO	
Matey1985 [14], 10.1063/1.334506	NO	NO	NO	C
Arakawa2001 [15], 10.1116/1.1379796	NO	NO	NO	V

Continued on next page

Table S1 – continued from previous page

Reference in the Paper, DOI	Force detection method	Detecting second capacity gradient	High Freq. Sweep possible	Remarks
Lee2006 [16], 10.1088/0957-4484/17/5/054	NO	NO	NO	dC/dV
Lee2002 [17], 10.1063/1.1505655	NO	NO	NO	dC/dV
Isenbart2001 [18], 10.1007/s003390100793	NO	NO	NO	dC/dV
Kopanski1998 [19], 10.1063/1.121397	NO	NO	NO	C
Casuso2007 [20], 10.1063/1.2767979	NO	NO	NO	C
Biberger2008 [21], 10.1016/j.microrel.2008.06.013	NO	NO	NO	dC/dV
Smoliner2001 [22], 10.1063/1.1415044	NO	NO	NO	dC/dV
Brezna2003 [23] 10.1063/1.1628402	NO	NO	NO	dC/dV
Giannazzo2006 [24], 10.1116/1.2151907	NO	NO	NO	C
Brezna2006 [25], 10.1063/1.2189030	NO	NO	NO	dC/dV
Futscher2019 [26], 10.1039/C9MH00445A	NO	NO	NO	C

Continued on next page

Table S1 – continued from previous page

Reference in the Paper, DOI	Force detection method	Detecting second capacity gradient	High Freq. Sweep possible	Remarks
Kopanski1997 [27], 10.1016/S0921-5107(96)01797-7	NO	NO	NO	dC/dV
Kopanski1996a [28], 10.1116/1.588455	NO	NO	NO	
Goto1998 [29], 10.1063/1.368617	NO	NO	NO	C
DeVoogd2017 [30], 10.1016/j.ultramic.2017.05.009	NO	NO	NO	C
Hiranaga2019 [31], 10.1063/1.5097906	NO	NO	NO	
Kobayashi2002 [32], 10.1063/1.1510582	YES	NO	NO	dC/dV
Martin1988 [33], 10.1063/1.99224	YES	NO	NO	
Abraham1991 [34], 10.1116/1.585536	YES	NO	NO	
Gramse2009 [35], 10.1088/0957-4484/20/39/395702	YES	NO	NO	
Fumagalli2010 [36], 10.1063/1.3427362	YES	NO	NO	
Fumagalli2012 [37], 10.1038/nmat3369	YES	NO	NO	

Continued on next page

Table S1 – continued from previous page

**Reference in the Paper, DOI**	**Force detection method**	**Detecting second capacity gradient**	**High Freq. Sweep possible**	**Remarks**
Gramse2012 [38], 10.1063/1.4768164	YES	NO	YES	
Gramse2013 [39], 10.1016/j.bpj.2013.02.011	YES	NO	NO	
Kimura2003 [40], 10.1016/S0169-4332(02)01486-1	YES	NO	NO	dC/dV
Henning1996 [41], 10.1016/S0921-5107(96)01688-1	YES	NO	NO	
Checa2021 [42], 10.1063/5.0078034	YES	NO	NO	
Li1998 [43], 10.1103/PhysRevB.57.9225	YES	NO	NO	
Gil2003 [44], 10.1088/0957-4484/14/2/345	YES	NO	NO	
Fukuzawa2020 [45], 10.1063/1.5127219	YES	NO	NO	dC/dV
Izumi2023 [46], 10.3762/bjnano.14.18	YES	NO	NO	
Cherniavskaya2003 [47], 10.1021/jp0265438	YES	NO	NO	
Crider2007a [48], 10.1063/1.2753539	YES	YES	NO	low freq. sweeps

Continued on next page

Table S1 – continued from previous page

**Reference in the Paper, DOI**	**Force detection method**	**Detecting second capacity gradient**	**High Freq. Sweep possible**	**Remarks**
Cadena2016 [49], 10.1021/jp0265438	YES	NO	YES	
Riedel2010 [50], 10.1103/PhysRevE.81.010801	YES	YES	NO	lift mode, freq. tracking
Gramse2019 [51], 10.1039/C8NR05880F	YES	YES	YES*	*: phase modulation
Gramse2020 [52], 10.1038/s41928-020-0450-8	YES	YES	YES§	§: limited to 1 MHz
Our Method: MFH-EFM	YES	YES	YES	dC/dV possible

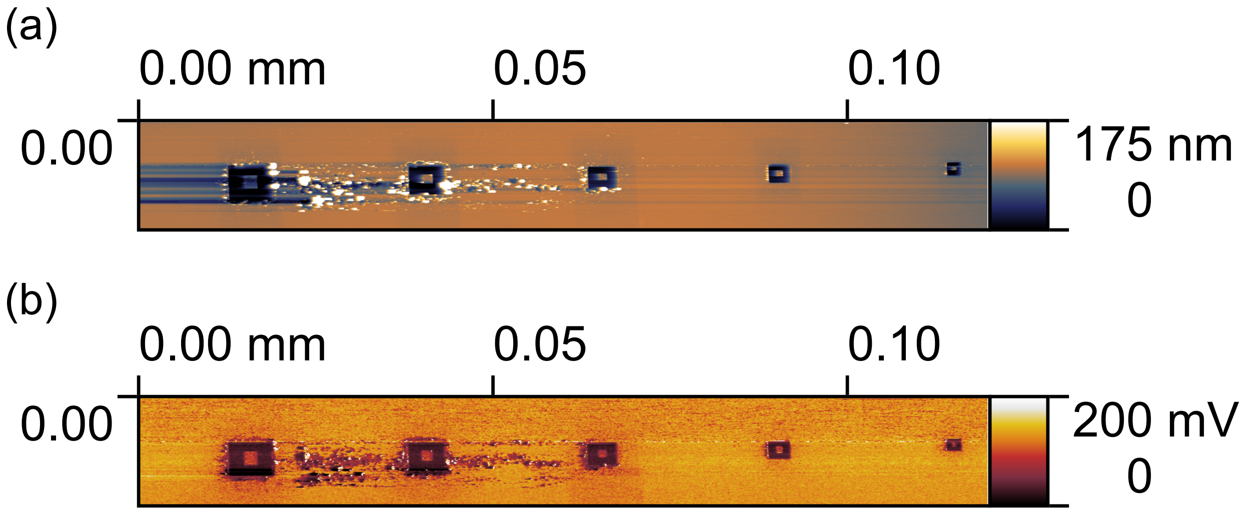

Figure S2: Raw data taken from the microcapacitors shown in Figure 2 in MFH-EFM mode. (a) The topography and (b) the A_{det} from the measurement. The image is not converted into C'' values but the relation is the same.

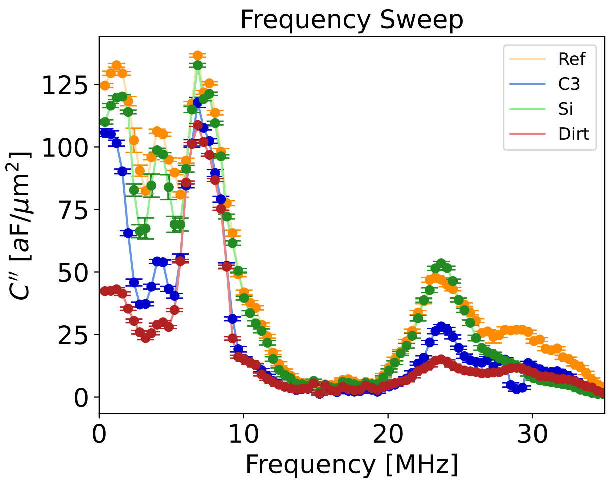

Figure S3: Non-normalized data of the comparison of the C'' frequency sweep shown in Figure 6 on the four spots while in MFH-EFM (see Equation 12). This was conducted with the μmasch's HQ:NSC18/Pt cantilever.

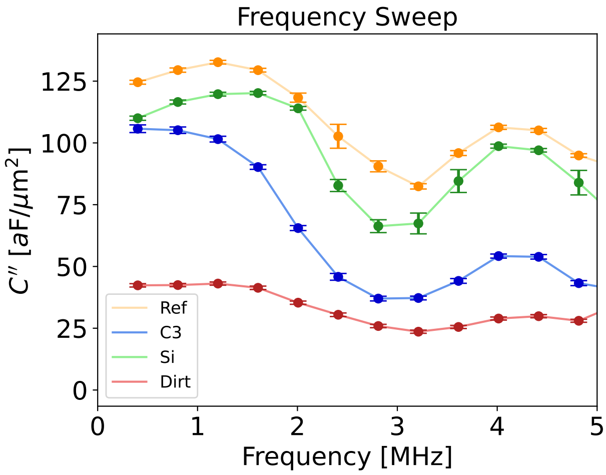

Figure S4: Zoom of the non-normalized data from the comparison of the C'' frequency sweep shown in Figure 6 on the four spots while in MFH-EFM (see Equation 12). This was conducted with the μmasch's HQ:NSC18/Pt cantilever.

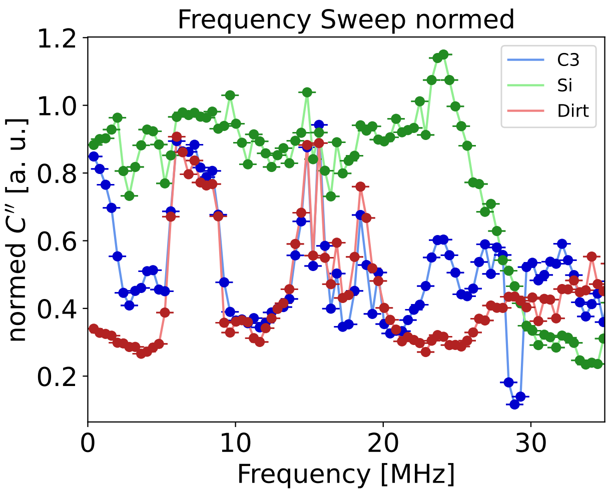

Figure S5: Normalized data of the C'' frequency sweep shown in Figure 6 on the three spots while in MFH-EFM (see Equation 12). This was conducted with the μmasch's HQ:NSC18/Pt cantilever.

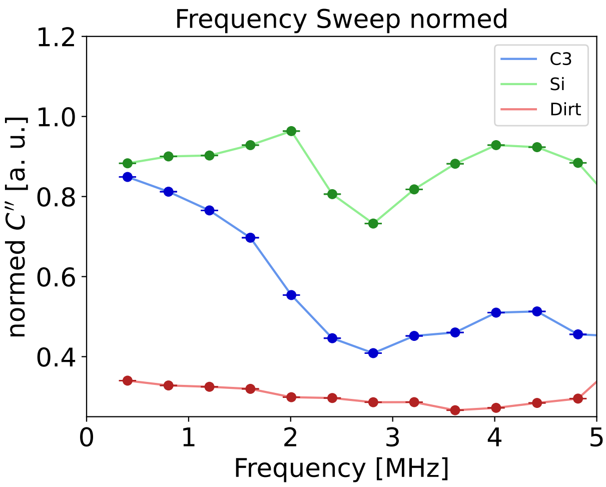

Figure S6: Zoomed and normalized data of the C'' frequency sweep shown in Figure 6 on the three spots while in MFH-EFM (see Equation 12). This was conducted with the μmasch's HQ:NSC18/Pt cantilever.

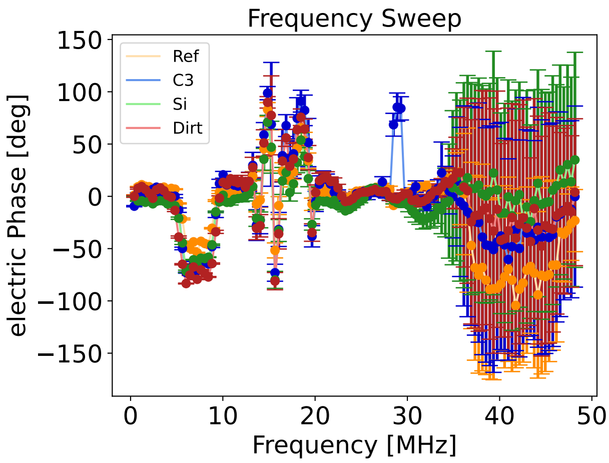

Figure S7: Non-normalized data of the phase signal φ spectra of the comparison from the C'' frequency sweep shown in Figure 6 on the four spots while in MFH-EFM (see Equation 12). This was conducted with the μmasch's HQ:NSC18/Pt cantilever.

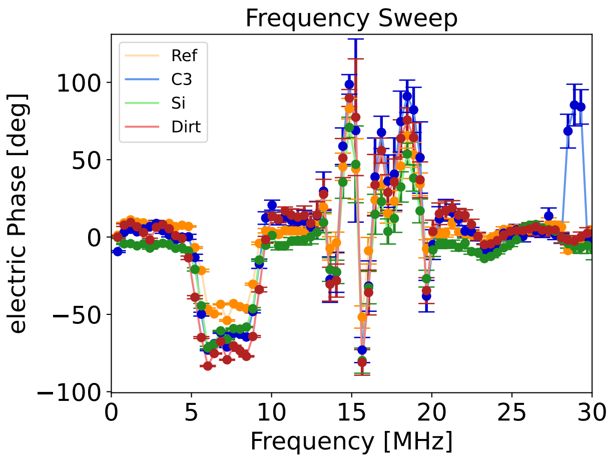

Figure S8: Zoom of the non-normalized data of the phase signal φ spectra of the comparison from the C'' frequency sweep shown in Figure 6 on the four spots while in MFH-EFM (see Equation 12). This was conducted with the μmasch's HQ:NSC18/Pt cantilever.

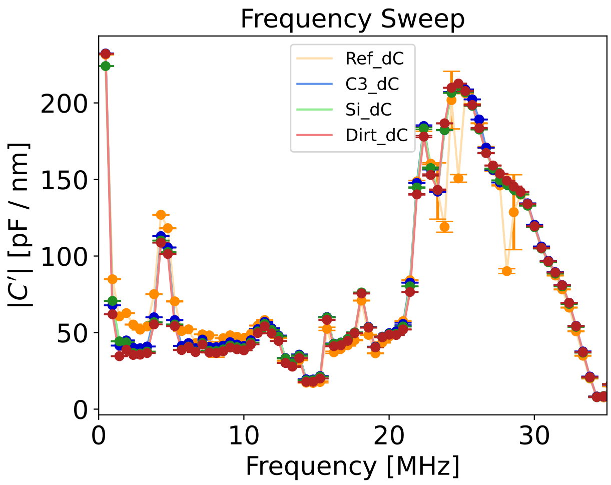

Figure S9: Non-normalized data of the comparison of the C' frequency sweep shown in Figure 6 on the four spots while in SF-EFM mode (see Equation 13). This was conducted with the μmasch's HQ:NSC18/Pt cantilever.

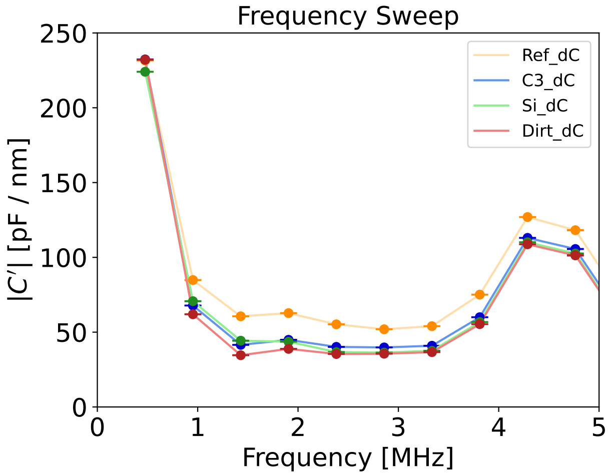

Figure S10: Zoom of the non-normalized data of the comparison of the C' frequency sweep shown in Figure 6 on the four spots while in SF-EFM mode (see Equation 13). This was conducted with the μmasch's HQ:NSC18/Pt cantilever.

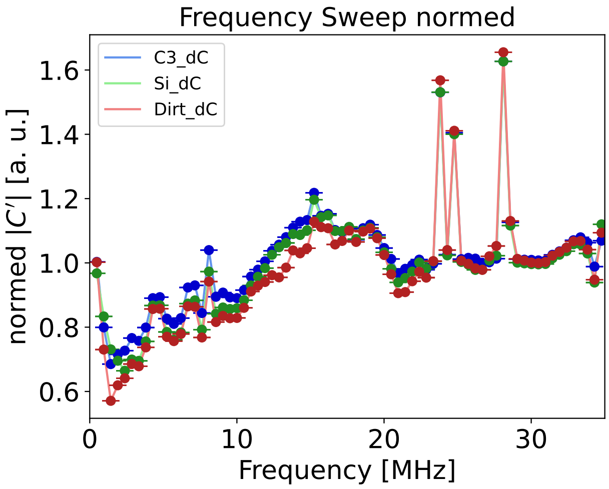

Figure S11: Normalized data of the C' frequency sweep shown in Figure 6 on the three spots while in SF-EFM mode (see Equation 13). This was conducted with the μmasch's HQ:NSC18/Pt cantilever.

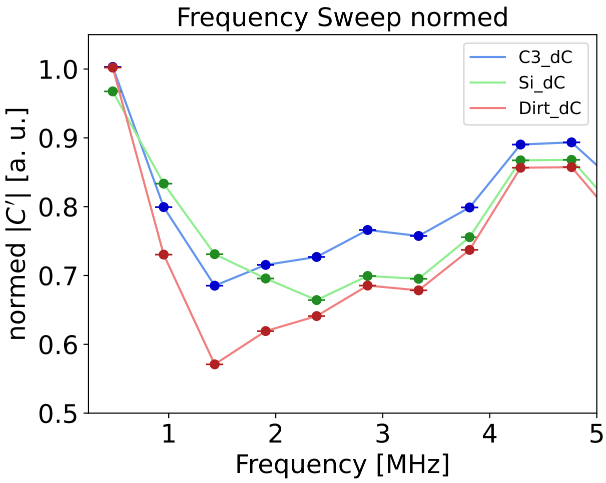

Figure S12: Zoomed and normalized data of the C' frequency sweep shown in Figure 6 on the three spots while in SF-EFM mode (see Equation 13). This was conducted with the μmasch's HQ:NSC18/Pt cantilever.

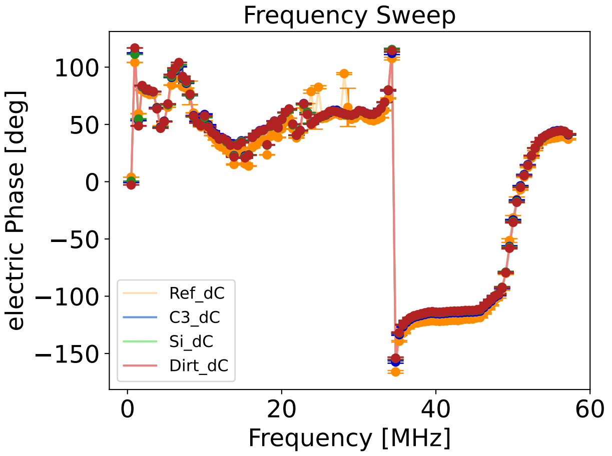

Figure S13: Non-normalized data of the phase signal φ spectra of the comparison of the C' frequency sweep shown in Figure 6 on the four spots while in SF-EFM mode (see Equation 13). This was conducted with the μmasch's HQ:NSC18/Pt cantilever.

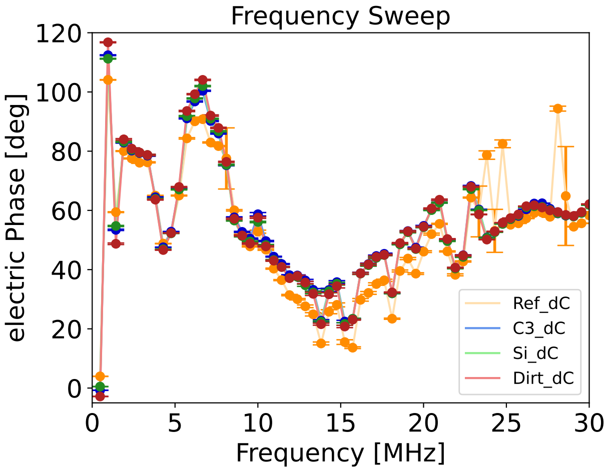

Figure S14: Zoom of the non-normalized data of the phase signal φ spectra of the comparison of the C' frequency sweep shown in Figure 6 on the four spots while in SF-EFM mode (see Equation 13). This was conducted with the μmasch's HQ:NSC18/Pt cantilever.

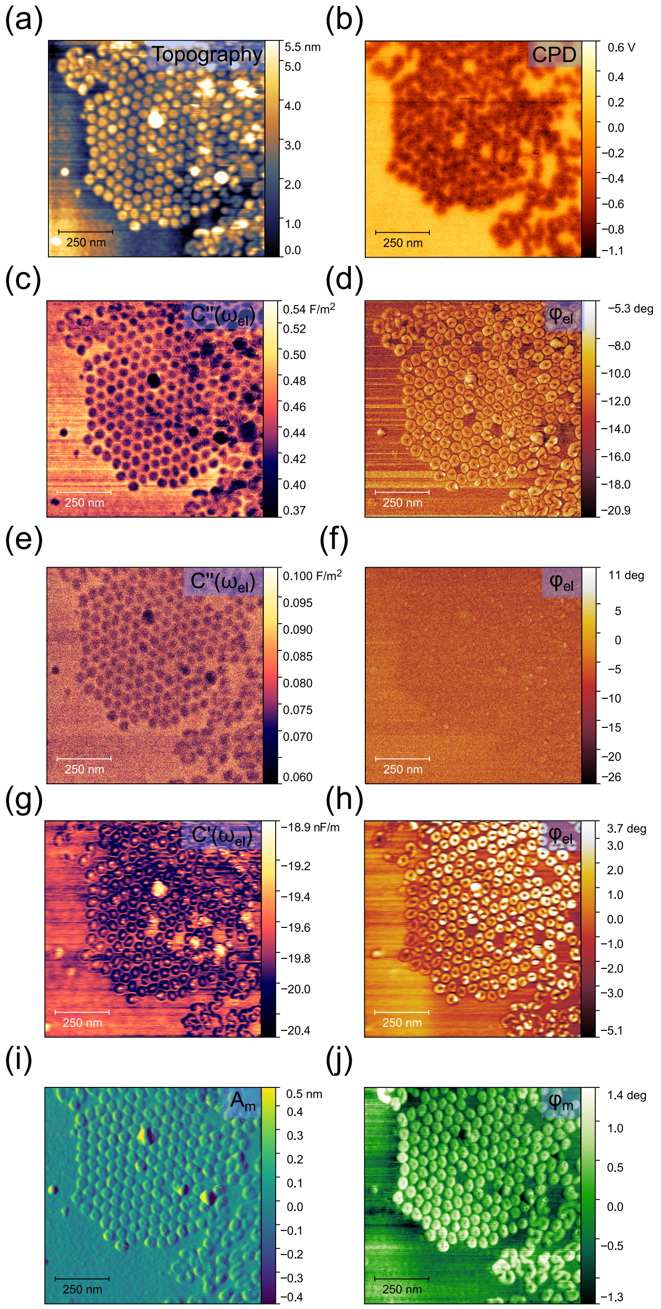

Figure S15: Full version of the MFH-EFM pictures given in Figure 7. MFH-EFM pictures made on F14H20 with (a) the topography, (b) the contact potential difference (CPD) picture, (c) the C'' picture detected at $\omega_{m,2}$, while excitation took place at frequencies 1.59 and 1.98 MHz (d) electric phase φ_{el} of the C'' signal detected at $\omega_{m,2}$, while excitation took place at frequencies 1.59 and 1.98 MHz, (e) the C'' picture detected at $\omega_{m,2}$, while excitation took place at frequencies 15.88 and 16.28 MHz, (f) electric phase φ_{el} of the C'' signal detected at $\omega_{m,2}$, while excitation took place at frequencies 15.88 and 16.28 MHz, (g) the C' picture detected at $\omega_{m,2}$, while excitation took place at 235.579 kHz, (h) electric phase φ_{el} of the C' signal detected at $\omega_{m,2}$, while excitation took place at frequency 235.579 kHz, (i) the picture of the mechanical amplitude at the resonance frequency of 74.580 kHz, and (j) the picture of the mechanical phase at the resonance frequency of 74.580 kHz. This was conducted with the μmasch's HQ:NSC18/Pt Cantilever.

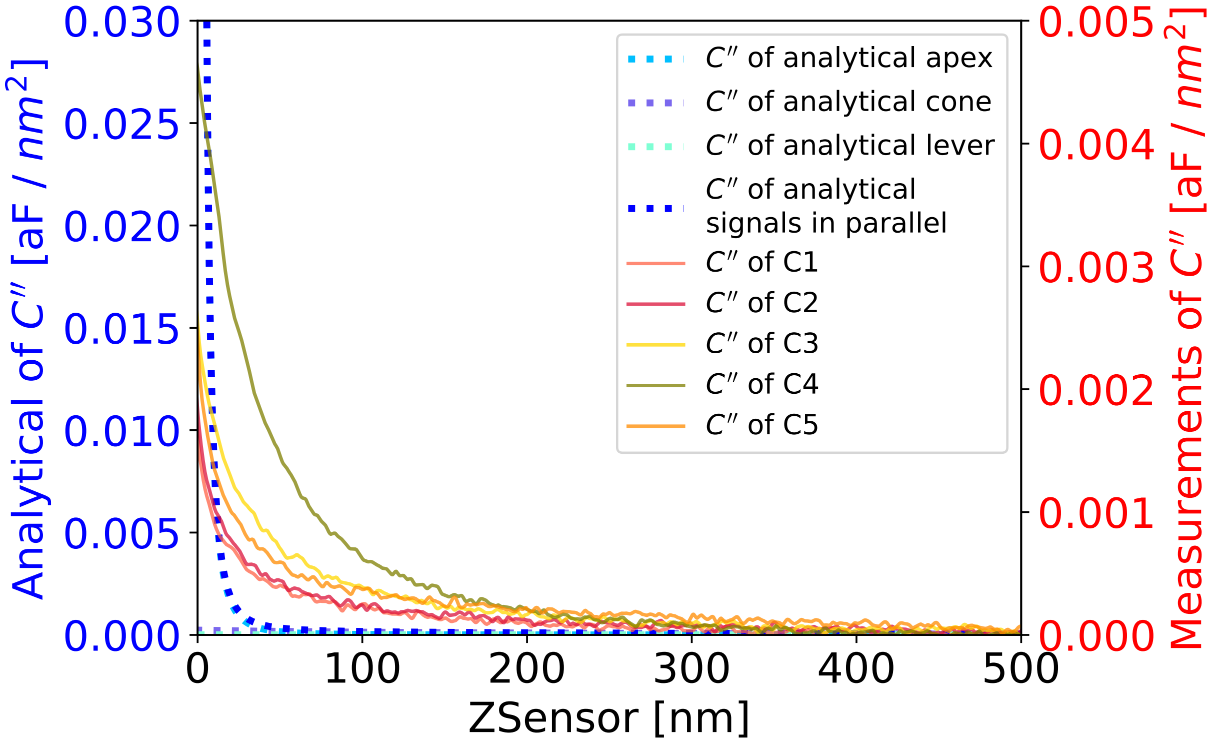

Figure S16: A comparison of the measured C'' values on various capacitors, as shown in Figure 2, is presented. The measurements, performed using the NuNano SPARK 70 Pt cantilever (solid lines), are contrasted with the theoretical contributions of the respective components to the first numerical derivative C' of the capacitance (dotted lines) as a function of the tip-to-sample distance, z For the theoretical calculations, the properties of the NuNano SPARK 70 Pt cantilever ($w = 30\,\mu\text{m}$, $l = 225\,\mu\text{m}$, $\alpha = 11\,\text{deg}$, $h = 12\,\mu\text{m}$, $\theta = 25\,\text{deg}$, $r = 18\,\text{nm}$, $\delta = 3.7 \cdot 10^{-7}$) with an mechanical amplitude of $A_\text{m} = 10\,\text{nm}$, an excitation voltage of $V_\text{AC} = 2\,\text{V}$, and a total amount of calculated points of 100,000, was used for these.

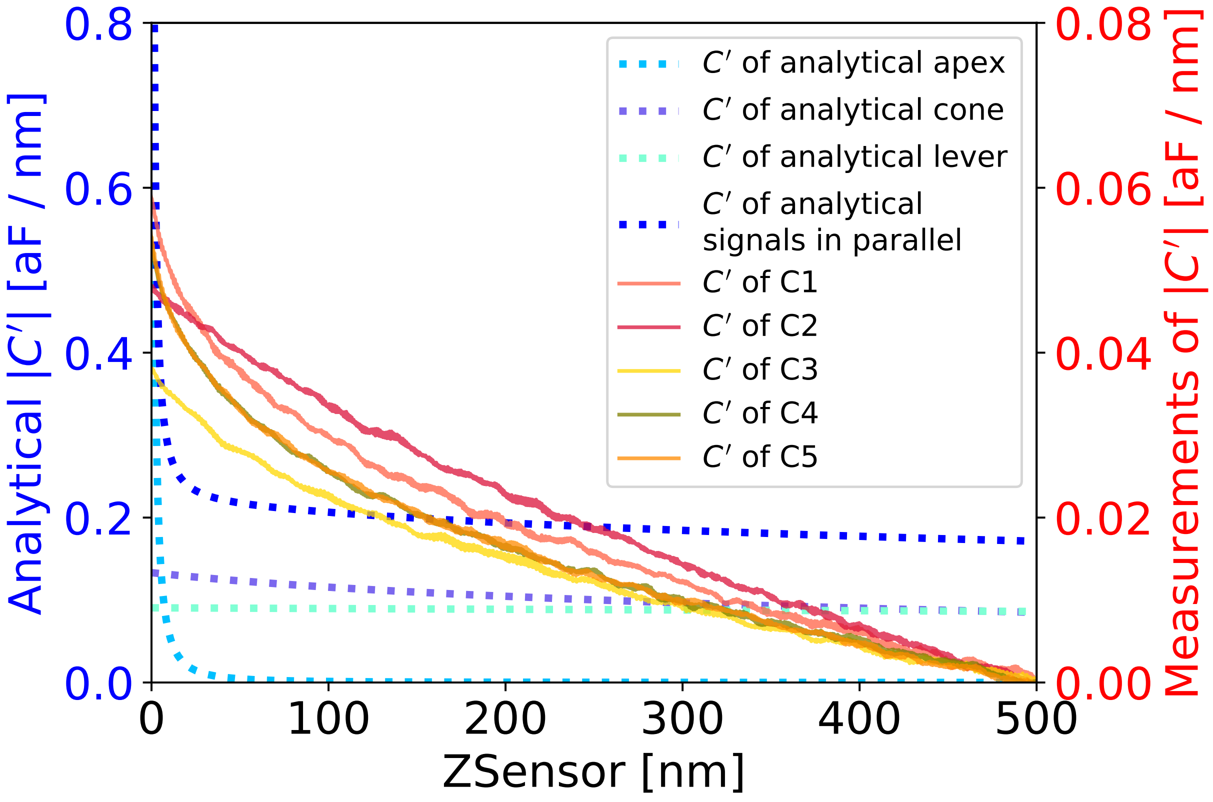

Figure S17: A comparison of the measured C' values on various capacitors, as shown in Figure 2, is presented. The measurements, performed using the NuNano SPARK 70 Pt cantilever (solid lines), are contrasted with the theoretical contributions of the respective components to the first numerical derivative C' of the capacitance (dotted lines) as a function of the tip-to-sample distance, z. For the theoretical calculations, the properties of the NuNano SPARK 70 Pt cantilever ($w = 30\,\mu$m, $l = 225\,\mu$m, $\alpha = 11$ deg, $h = 12\,\mu$m, $\theta = 25$ deg, $r = 18$ nm, $\delta = 3.7 \cdot 10^{-7}$) with an mechanical amplitude of $A_{\mathrm{m}} = 10$ nm, an excitation voltage of $V_{\mathrm{AC}} = 2$ V, and a total amount of calculated points of 100,000, was used for these.

References

1. Garrett, J. L.; Munday, J. N. *Nanotechnology* **2016**, *27* (24), 14. doi:10.1088/0957-4484/27/24/245705.

2. Axt, A.; Hermes, I. M.; Bergmann, V. W.; Tausendpfund, N.; Weber, S. A. L. *Beilstein J. Nanotechnol.* **2018**, *9* (1), 1809–1819. doi:10.3762/bjnano.9.172.

3. Jaensch, S.; Schmidt, H.; Grundmann, M. *Phys. B Condens. Matter* **2006**, *376-377* (1), 913–915. doi:10.1016/j.physb.2005.12.227.

4. Tran, T.; Oliver, D.; Thomson, D.; Bridges, G. Sub-zeptofarad sensitivity scanning capacitance microscopy. In *IEEE CCECE2002. Can. Conf. Electr. Comput. Eng. Conf. Proc. (Cat. No.02CH37373)*; IEEE, 2002; pp 455–459. doi:10.1109/CCECE.2002.1015268.

5. Raineri, V.; Giannazzo, F. *Solid State Phenom.* **2001**, *78-79*, 425–0. doi:10.4028/www.scientific.net/SSP.78-79.425.

6. Barrett, R. C.; Quate, C. F. *J. Appl. Phys.* **1991**, *70* (5), 2725–2733. doi:10.1063/1.349388.

7. Yamamoto, T.; Suzuki, Y.; Hiroyuki Sugimura, H. S.; Nobuyuki Nakagiri, N. N. *Jpn. J. Appl. Phys.* **1996**, *35* (6S), 3793. doi:10.1143/JJAP.35.3793.

8. Goto, K.; Hane, K. *Rev. Sci. Instrum.* **1997**, *68* (1), 120–123. doi:10.1063/1.1147749.

9. Goto, K.; Hane, K. <title>Tapping mode scanning capacitance microscopy: feasibility of quantitative capacitance measurement</title>. In *Micromach. Imaging*; Michalske, T. A., Wendman, M. A., Eds.; 1997; pp 84–91. doi:10.1117/12.271216.

10. Fumagalli, L.; Ferrari, G.; Sampietro, M.; Casuso, I.; Martínez, E.; Samitier, J.; Gomila, G. *Nanotechnology* **2006**, *17* (18), 4581–4587. doi:10.1088/0957-4484/17/18/009.

11. Fumagalli, L.; Ferrari, G.; Sampietro, M.; Gomila, G. *Appl. Phys. Lett.* **2007**, *91* (24), 243110. doi:10.1063/1.2821119.

12. Gomila, G.; Toset, J.; Fumagalli, L. *J. Appl. Phys.* **2008**, *104* (2), 024315. doi:10.1063/1.2957069.

13. Fumagalli, L.; Ferrari, G.; Sampietro, M.; Gomila, G. *Nano Lett.* **2009**, *9* (4), 1604–1608. doi:10.1021/nl803851u.

14. Matey, J. R.; Blanc, J. *J. Appl. Phys.* **1985**, *57* (5), 1437–1444. doi:10.1063/1.334506.

15. Arakawa, H.; Nishitani, R. *J. Vac. Sci. Technol. B Microelectron. Nanom. Struct. Process. Meas. Phenom.* **2001**, *19* (4), 1150–1153. doi:10.1116/1.1379796.

16. Lee, D. T.; Pelz, J. P.; Bhushan, B. *Nanotechnology* **2006**, *17* (5), 1484–1491. doi:10.1088/0957-4484/17/5/054.

17. Lee, D. T.; Pelz, J. P.; Bhushan, B. *Rev. Sci. Instrum.* **2002**, *73* (10), 3525–3533. doi:10.1063/1.1505655.

18. Isenbart, J.; Born, A.; Wiesendanger, R. *Appl. Phys. A Mater. Sci. Process.* **2001**, *72* (S2), S243–S251. doi:10.1007/s003390100793.

19. Kopanski, J. J.; Mayo, S. *Appl. Phys. Lett.* **1998**, *72* (19), 2469–2471. doi:10.1063/1.121397.

20. Casuso, I.; Fumagalli, L.; Gomila, G.; Padrós, E. *Appl. Phys. Lett.* **2007**, *91* (6), 063111. doi:10.1063/1.2767979.

21. Biberger, R.; Benstetter, G.; Schweinboeck, T.; Breitschopf, P.; Goebel, H. *Microelectron. Reliab.* **2008**, *48* (8-9), 1339–1342. doi:10.1016/j.microrel.2008.06.013.

22. Smoliner, J.; Basnar, B.; Golka, S.; Gornik, E.; Löffler, B.; Schatzmayr, M.; Enichlmair, H. *Appl. Phys. Lett.* **2001**, *79* (19), 3182–3184. doi:10.1063/1.1415044.

23. Brezna, W.; Schramboeck, M.; Lugstein, A.; Harasek, S.; Enichlmair, H.; Bertagnolli, E.; Gornik, E.; Smoliner, J. *Appl. Phys. Lett.* **2003**, *83* (20), 4253–4255. doi:10.1063/1.1628402.

24. Giannazzo, F.; Raineri, V.; Mirabella, S.; Impellizzeri, G.; Priolo, F.; Fedele, M.; Mucciato, R. *J. Vac. Sci. Technol. B Microelectron. Nanom. Struct. Process. Meas. Phenom.* **2006**, *24* (1), 370–374. doi:10.1116/1.2151907.

25. Brezna, W.; Fischer, M.; Wanzenboeck, H. D.; Bertagnolli, E.; Smoliner, J. *Appl. Phys. Lett.* **2006**, *88* (12), 122116. doi:10.1063/1.2189030.

26. Futscher, M. H.; Lee, J. M.; McGovern, L.; Muscarella, L. A.; Wang, T.; Haider, M. I.; Fakharuddin, A.; Schmidt-Mende, L.; Ehrler, B. *Mater. Horizons* **2019**, *6* (7), 1497–1503. doi:10.1039/C9MH00445A.

27. Kopanski, J. J.; Marchiando, J. F.; Lowney, J. R. *Mater. Sci. Eng. B* **1997**, *44* (1-3), 46–51. doi:10.1016/S0921-5107(96)01797-7.

28. Kopanski, J. J.; Marchiando, J. F.; Lowney, J. R. *J. Vac. Sci. Technol. B Microelectron. Nanom. Struct. Process. Meas. Phenom.* **1996**, *14* (1), 242–247. doi:10.1116/1.588455.

29. Goto, K.; Hane, K. *J. Appl. Phys.* **1998**, *84* (8), 4043–4048. doi:10.1063/1.368617.

30. de Voogd, J.; van Spronsen, M.; Kalff, F.; Bryant, B.; Ostojić, O.; den Haan, A.; Groot, I.; Oosterkamp, T.; Otte, A.; Rost, M. *Ultramicroscopy* **2017**, *181*, 61–69. doi:10.1016/j.ultramic.2017.05.009.

31. Hiranaga, Y.; Cho, Y. *Rev. Sci. Instrum.* **2019**, *90* (8), 1–12. doi:10.1063/1.5097906.

32. Kobayashi, K.; Yamada, H.; Matsushige, K. *Appl. Phys. Lett.* **2002**, *81* (14), 2629–2631. doi:10.1063/1.1510582.

33. Martin, Y.; Abraham, D. W.; Wickramasinghe, H. K. *Appl. Phys. Lett.* **1988**, *52* (13), 1103–1105. doi:10.1063/1.99224.

34. Abraham, D. W.; Williams, C.; Slinkman, J.; Wickramasinghe, H. K. *J. Vac. Sci. Technol. B Microelectron. Nanom. Struct. Process. Meas. Phenom.* **1991**, *9* (2), 703–706. doi:10.1116/1.585536.

35. Gramse, G.; Casuso, I.; Toset, J.; Fumagalli, L.; Gomila, G. *Nanotechnology* **2009**, *20* (39), 395702. doi:10.1088/0957-4484/20/39/395702.

36. Fumagalli, L.; Gramse, G.; Esteban-Ferrer, D.; Edwards, M. A.; Gomila, G. *Appl. Phys. Lett.* **2010**, *96* (18), 183107. doi:10.1063/1.3427362.

37. Fumagalli, L.; Esteban-Ferrer, D.; Cuervo, A.; Carrascosa, J. L.; Gomila, G. *Nat. Mater.* **2012**, *11* (9), 808–816. doi:10.1038/nmat3369.

38. Gramse, G.; Edwards, M. A.; Fumagalli, L.; Gomila, G. *Appl. Phys. Lett.* **2012**, *101* (21), 213108. doi:10.1063/1.4768164.

39. Gramse, G.; Dols-Perez, A.; Edwards, M.; Fumagalli, L.; Gomila, G. *Biophys. J.* **2013**, *104* (6), 1257–1262. doi:10.1016/j.bpj.2013.02.011.

40. Kimura, K.; Kobayashi, K.; Yamada, H.; Matsushige, K. *Appl. Surf. Sci.* **2003**, *210* (1-2), 93–98. doi:10.1016/S0169-4332(02)01486-1.

41. Henning, A.; Hochwitz, T. *Mater. Sci. Eng. B* **1996**, *42* (1-3), 88–98. doi:10.1016/S0921-5107(96)01688-1.

42. Checa, M.; Neumayer, S. M.; Susner, M. A.; McGuire, M. A.; Maksymovych, P.; Collins, L. *Appl. Phys. Lett.* **2021**, *119* (25), 252905. doi:10.1063/5.0078034.

43. Li, Z.-y.; Gu, B.-y.; Yang, G.-z. *Phys. Rev. B* **1998**, *57* (15), 9225–9233. doi:10.1103/PhysRevB.57.9225.

44. Gil, A.; Colchero, J.; G mez Herrero, J.; Bar, A. M. *Nanotechnology* **2003**, *14* (2), 332–340. doi:10.1088/0957-4484/14/2/345.

45. Fukuzawa, R.; Takahashi, T. *Rev. Sci. Instrum.* **2020**, *91* (2), 023702. doi:10.1063/1.5127219.

46. Izumi, R.; Miyazaki, M.; Li, Y. J.; Sugawara, Y. *Beilstein J. Nanotechnol.* **2023**, *14*, 175–189. doi:10.3762/bjnano.14.18.

47. Cherniavskaya, O.; Chen, L.; Weng, V.; Yuditsky, L.; Brus, L. E. *J. Phys. Chem. B* **2003**, *107* (7), 1525–1531. doi:10.1021/jp0265438.

48. Crider, P. S.; Majewski, M. R.; Zhang, J.; Oukris, H.; Israeloff, N. E. *Appl. Phys. Lett.* **2007**, *91* (1), 2–5. doi:10.1063/1.2753539.

49. Cadena, M. J.; Sung, S. H.; Boudouris, B. W.; Reifenberger, R.; Raman, A. *ACS Nano* **2016**, *10*, 4062–4071. doi:10.1021/acsnano.5b06893. PMID: 26972782

50. Riedel, C.; Arinero, R.; Tordjeman, P.; Lévêque, G.; Schwartz, G. A.; Alegria, A.; Colmenero, J. *Phys. Rev. E* **2010**, *81* (1), 010801. doi:10.1103/PhysRevE.81.010801.

51. Gramse, G.; Schönhals, A.; Kienberger, F. *Nanoscale* **2019**, *11* (10), 4303–4309. doi:10.1039/C8NR05880F.

52. Gramse, G.; Kölker, A.; Škereň, T.; Stock, T. J. Z.; Aeppli, G.; Kienberger, F.; Fuhrer, A.; Curson, N. J. *Nat. Electron.* **2020**, *3* (9), 531–538. doi:10.1038/s41928-020-0450-8.
